# TZM Mo alloy behaves superb as biodegradable metal for bone-fracture healing intramedullary nail implant

**DOI:** 10.1016/j.mtbio.2026.102794

**Published:** 2026-01-12

**Authors:** Junyu Qian, Yukun Zhou, Zhenhai Xie, Jinjing Liu, Ping Li, Wenjie Tao, Yuanhao Wang, Fei Gao, Hui Zeng, Deli Wang, Haotian Qin, Yingqi Chen, Guojiang Wan

**Affiliations:** a Institute of Biomedical Engineering, College of Medicine, Key Laboratory of Advanced Technologies of Materials, Ministry of Education, Southwest Jiaotong University, Chengdu, 610031, China; b National & Local Joint Engineering Research Centre of Orthopaedic Biomaterials, Department of Bone & Joint Surgery, Peking University Shenzhen Hospital, Shenzhen, 518036, China; c Shenzhen Peking University-The Hong Kong University of Science and Technology Medical Center, Peking University Shenzhen Hospital, Shenzhen, 518036, China; d School and Hospital of Stomatology, Guangdong Engineering Research Center of Oral Restoration and Reconstruction, Guangzhou Medical University, Guangzhou, China; e Department of Orthopedics, Medical Innovation Technology Transformation Center of Shenzhen Second People's Hospital, Shenzhen Second People's Hospital, Shenzhen, Guangdong, China; f Medical Innovation Technology Transformation Center of Shenzhen Second People's Hospital, Shenzhen Second People's Hospital, Shenzhen, Guangdong, China

**Keywords:** Biodegradable metals, TZM Mo alloy, Intramedullary nail, Bone fracture healing, Angiogenesis & osteogenesis coupling

## Abstract

Bone fracture repair, particularly assisted by load-bearing implants, faces tough clinical challenges, necessitating novel biomaterials that are mechanically strong, biocompatible and biodegradable to achieve effective healing. Metallic molybdenum (Mo) has shown promise in this regard, whereas little has been done with considering its alloys that are more advantageous on many aspects over its pure counterpart. Herein, we demonstrate that the TZM Mo alloy (namely Titanium-Zirconium-Molybdenum, also Mo-Ti-Zr) performed superb efficacy in the repair of rat femoral fractures with its intramedullary nails (IMNs) prototype product even as compared with pure Mo. The TZM alloy had superior mechanical strength and more uniform degradation than Mo, meeting the requirements for next-generation biodegradable IMNs. Moreover, the *in vitro* assays verified the TZM promoted adhesion, migration and proliferation of endothelial cells and bone marrow mesenchymal stem cells and elicited no toxicity. Molecular expression results revealed the TZM may enhance angiogenesis by activating Wnt/β-catenin signaling and facilitated bone formation by up-regulating osteogenic genes via PI3K–Akt, MAPK–ERK, and cAMP–PKA pathways. More important, TZM-based IMNs achieved nearly complete fracture healing at 12 weeks in a rat femoral fracture model. Thus, the TZM Mo alloy holds super potential for clinical translation.

## Graphical abstract

**Figure:**
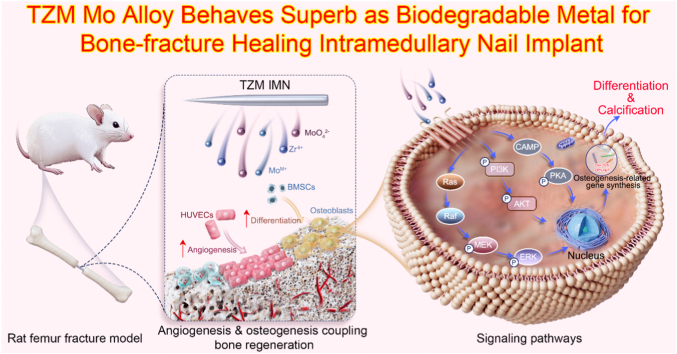

## 1 Introduction

Bone fractures are a common clinical orthopedic condition frequently seen following traumatic injuries, being a global public health issue that imposes a serious economic burden [1,2]. The incidence of bone fractures increases with age; according to the Global Burden of Diseases, Injuries, and Risk Factors Study (GBD) report, new fracture cases worldwide have reached approximately 170 million new cases annually [2]. Bone fracture healing is a complex physiological process that involves a series of events at multiple levels [3]. It is estimated that 5–10 % of bone fractures fail to heal properly, leading to delayed union or non-union problem and related complications, a further threat to the patients’ health and lives [4].

For bone fracture treatments, internal fixation is an indispensable step realized by surgical implantation of devices such as bone plates, Kirschner wires, screws, and intramedullary nails (IMNs) to support bone healing [3]. Although the method has been well-established, it is not without problems as the existing materials are all permanent materials. The typical metallic materials such as Ti and its alloys, stainless steel (SS), and CoCrMo alloys, currently used for these implants are all considered to provide ever-lasting mechanical support and supposed to be inert in the physiological circumstance [3,5]. However, these inert metals have inherent limitations: they often require secondary surgery for removal usually because of chronic inflammation due to their long-term presence, and lack bio-activity to promote bone regeneration of fracture repairing [6,7]. These drawbacks have driven interest in biodegradable metallic biomaterials that can fully degrade in coordination with the healing process, leaving no harmful residues.

Representative biodegradable metals (BMs) like magnesium (Mg), zinc (Zn) and their alloys have been widely exploited for internal fixation implants thanks to their good biodegradability and bio-compatibility/activities [[8], [9]], but face still big challenges in their clinical translation into such load-bearing implants. Mg-based implants, despite their initial clinical success, suffer from overly rapid degradation and hydrogen gas release, causing premature failure and local bone resorption [10,11]. Zn and its alloys offer improved degradation stability but face issues such as strain-softening and cytotoxicity at high Zn^2+^ concentrations [12]. More important, neither Mg nor Zn-based implants can easily overcome shortcomings like poor mechanical properties and uncontrollable ion release even through surface modification alone [13,14]. Therefore, more advanced new BMs are still urgently needed for temporary biomedical implants especially with highly load-bearing demand.

Very recently, molybdenum (Mo) emerged as a promising new BM candidate with surprisingly good biodegradability and biocompatibilities along with its obviously advantageous mechanical properties over other BMs as above-mentioned [15]. Metallic Mo exhibits ultrahigh mechanical strength and stiffness superior to traditional biomaterials [16,17], coupled with a uniform degradation pattern and favorable radiopacity [18,19]. Its degradation products (MoO_3_, molybdate salts) are biocompatible [20,21], excreted efficiently without organ accumulation in animal models [22,23], and promote osteogenesis [24,25]. As a trace element involved in essential enzymatic reactions [26,27], Mo also modulates mitochondrial function and macrophage immune responses [28], creating a regenerative microenvironment [29,30]. Hence, metallic Mo stands out as a great potential BM for bone fracture fixation and support.

Nevertheless, up to date most of the attentions have been paid on pure Mo as a new BM. Pure Mo brings inevitably with itself some weaknesses such as falling short of easily micro-structure adjustment and therefore comprehensive mechanical properties, among others. Some commercial Mo alloys have already been well developed for advanced industrial applications and they offer a plenty of implications for biomedical usage, whereas various issues require further exploration. For instance, the Mo-Ti-Zr alloy, also called TZM (Titanium-Zirconium-Molybdenum), where the alloying elements Ti: 0.4–0.6 wt%, Zr: 0.08–0.12 wt% and C: 0.02–0.03 wt% are all well-established in orthopedic applications, have obviously superior properties than pure Mo (etc. refined grain structure, higher grain boundary strength, and fewer grain boundary defects) [31]. This Mo alloy is supposed to be a more ideal BM especially for load-bearing bone implants albeit remains unexplored. Some investigations have been primarily conducted on the TZM (Mo-Ti-Zr) alloy for biomedical applications [31], whereas it was still postulated as non-degradable metallic biomaterials and evaluated only from a general point of view to find good cytocompatibility. To the best of our knowledge, no studies have yet examined the TZM alloy as biodegradable metal and not to mention validated it on a real implant and more practical circumstance. Research on its *in vitro* and *in vivo* corrosion/degradation behavior, osteogenic activity, regulation of bone regeneration, and the efficacy on a load-bearing implant such as IMNs in animal models like femoral fracture as well as the underlying biological mechanisms, remains scarce and of great necessity.

**Scheme 1:**
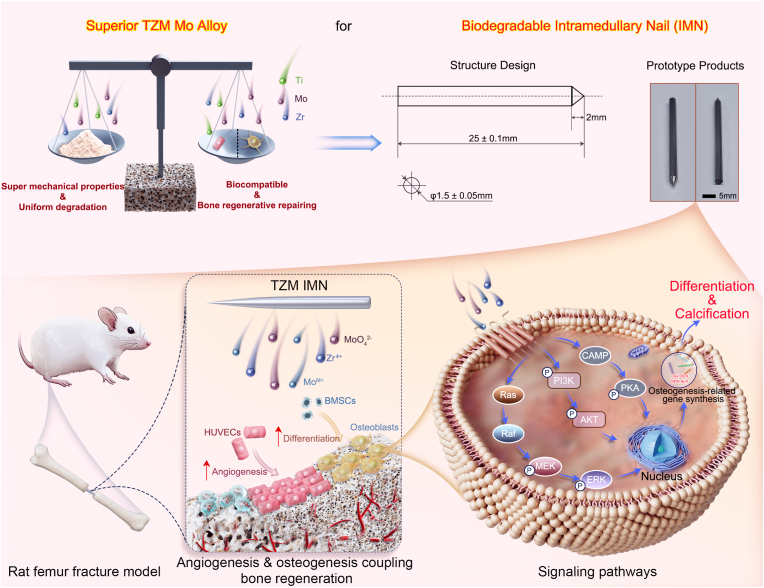
Schematic illustration of materials design, pro-osteogenesis and angiogenesis activity and potential molecular mechanism of Mo-Ti-Zr-based intramedullary nails.

Here in this study, the TZM alloy was systematically assessed by *in vitro* assays as well as *in vivo* experiments on its prototype product of IMNs. More specific, as shown in Scheme 1, the prototype TZM IMN products were made, and the adaptability of mechanical properties with the degradation process as well as angiogenesis & osteogenesis coupling bone regenerative activities of the TZM alloy were evaluated and compared with those of pure Mo and AZ31 Mg alloy (a commonly-investigated BM). The biological mechanisms were fathomed through transcription sequencing and verified by reverse transcription-quantitative polymerase chain reaction (RT-qPCR) and western blotting (WB) assay. Most important, the *in vivo* fracture healing efficacy of the TZM IMNs was validated in a rat femoral fracture model as compared with the pure Mo IMNs. Our findings in this work provide not only new insights in translating this commercially-available advanced industrial material directly into new biomedical usage, but also lay a theoretical foundation for adding the Mo alloys into the family of BMs to expand their spectrum of applications.

## 2 Results

### 2.1 Characterization

**Fig. 1:**
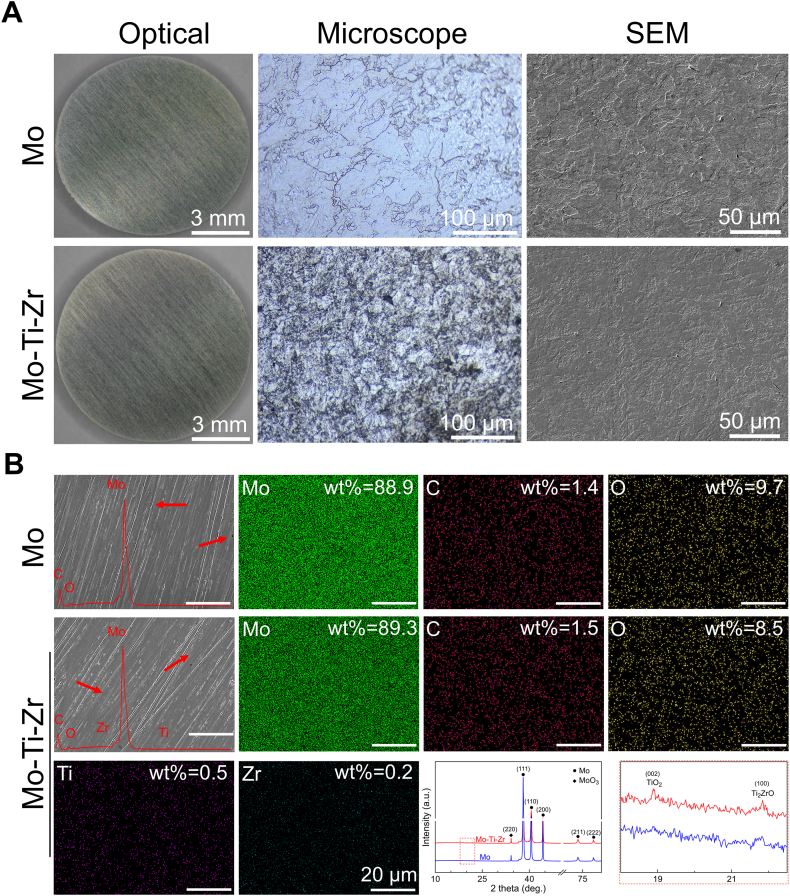
Materials characterization of Mo and Mo-Ti-Zr. (A) Representative surface optical images, metallography pictures, and SEM morphology. (B) EDS mapping and curves of Mo and Mo-Ti-Zr (green: Mo; red: C; yellow: O; violet: Ti; cyan: Zr) and XRD patterns. (For interpretation of the references to color in this figure legend, the reader is referred to the Web version of this article.)

The material properties of TZM alloy (Mo-Ti-Zr) were compared with those of pure Mo (Fig. 1). Although the macro-optical images depict the Mo-Ti-Zr appeared similar luster surface with Mo, its metallographic microscope image shows apparently more packed micro-structure morphology as compared to the latter (Fig. 1A). In more details, the Mo presents relatively big-size grains intermixed diversely separated by some clear cracks-like regions caused by a certain of loosely bonding at the grain boundaries after the powder sintering process, a phenomenon also reported in previous studies [21]. In comparison, the grains in the Mo-Ti-Zr alloy were significantly refined, bringing with themselves a more homogenous intermixture. The scanning electron microscopy (SEM) images further confirmed these results. Thus, the addition of Ti and Zr notably improves the microstructural properties of the Mo matrix. Energy dispersive spectroscopy (EDS) mapping results of the Mo-Ti-Zr and pure Mo showed a uniform distribution of Mo and O elements in both surfaces (Fig. 1B). Small amounts of Ti and Zr were also detected in the Mo-Ti-Zr alloy. In X-ray diffraction (XRD) patterns, both the Mo and the Mo-Ti-Zr alloy exhibited distinct diffraction peaks of Mo and an MoO_3_ peak. In the Mo-Ti-Zr alloy, characteristic peaks of TiO_2_ and ZrO_2_ were also observed, indicating that the addition of Ti and Zr not only altered the microstructure of the Mo matrix but also led to the formation of new oxide phases.

### 2.2 *In vitro* corrosion behavior of the Mo-Ti-Zr compared to pure Mo

**Fig. 2:**
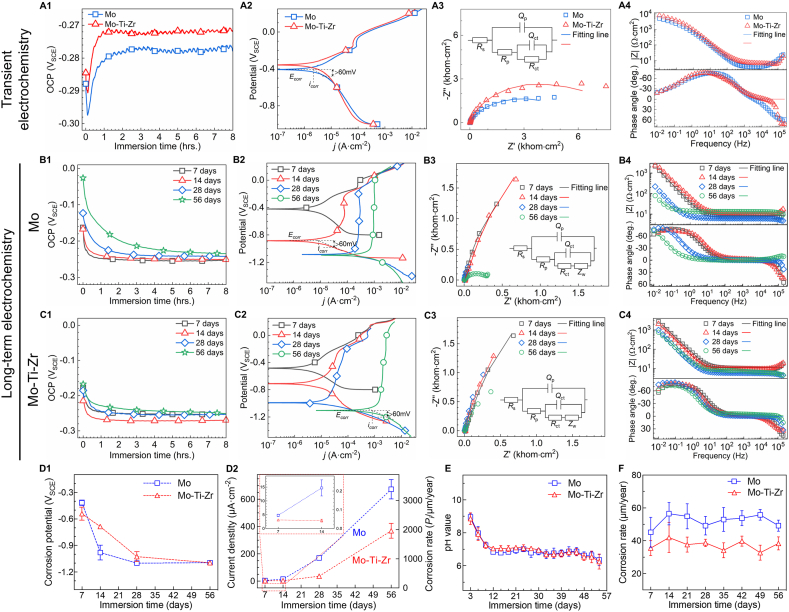
Transient and long-term (7, 14, 28, and 56 days) electrochemical measurements of Mo and Mo-Ti-Zr immersed in Hank's solution at semi-static condition at 37 °C. (A1) Open circuit potential (OCP) vs. time curves. (A2) Potentiodynamic polarization (PDP) curves. (A3) Nyquist plots (embedded with equivalent electron circuit; EEC). (A4) Bode-impedance and bode-phase angle spectra. (B1–B4) OCP, PDP, Nyquist, and bode plots of Mo at different immersion time points, respectively. (C1–C4) OCP, PDP, Nyquist, and bode plots of Mo-Ti-Zr at different immersion time points, respectively. (D1) Corrosion potential (*E*_corr_) of Mo and Mo-Ti-Zr obtained from PDP curves with the immersion time. (D2) Corrosion current density (*i*_corr_) of Mo-Ti-Zr and Mo obtained from PDP curves and the corresponding corrosion rate. (E) pH value of Hank's solution during the immersion process. (F) Corrosion rate of Mo and Mo-Ti-Zr calculated according to the weight loss method at different immersion time points.

Electrochemical tests and long-term immersion experiments (7, 14, 28, and 56 days) were conducted to investigate the corrosion and degradation behavior of the Mo-Ti-Zr and pure Mo in Hank's solution (Fig. 2). It shows that the open circuit potential (OCP) curve (Fig. 2A1) of the Mo-Ti-Zr was more positive than that of pure Mo, indicating higher thermodynamic stability. Both the Mo-Ti-Zr and pure Mo showed similar trends in their OCP curves, with a drop at the beginning of the test, followed by a gradual rise within the first hour, finally stabilizing at a constant level. The initial drop in potential might be due to the instability of the metallic surface upon contact with Hank's solution, a phenomenon also observed in our previous studies [21]. The increase in potential within the first hour is caused by the mixed potential due to anodic oxidation of Mo matrix and cathodic reduction of O_2_. Thereafter, the evolving formation of a MoO_2_ oxide film on the surface inhibited temporarily the reactions between the electrolyte and the substrate, resulting in a relatively stable interfacial potential [15,21]. In the potentiodynamic polarization (PDP) curves, the Mo-Ti-Zr had a higher corrosion potential (*E*_corr_) compared to the pure Mo (Fig. 2A2), consistent with the OCP curves. A passive region was observed in the anodic branches of both PDP curves, which may be associated with the more readily formation of surface oxides layer. The corrosion current density (*i*_corr_) was assessed by the Tafel extrapolation method (Table S1, Supplementary Materials). The Mo-Ti-Zr showed a slightly lower *i*_corr_ value than the pure Mo. The corrosion rates of the Mo-Ti-Zr and the Mo, calculated based on the formula *P*_*i*_ = 5.09*i*_corr_ (refer to Equation (1) in the Supplementary materials for details), were 14.30 ± 2.34 and 16.14 ± 1.37 μm/year, respectively. Electrochemical impedance spectroscopy (EIS) plots for the Mo-Ti-Zr and pure Mo (Fig. 2A3 and Fig. 2A4), specifically in Nyquist and Bode types, showed a slightly larger impedance value for the Mo-Ti-Zr compared to the pure Mo, indicating a higher corrosion resistance. In the Bode phase plots, peaks were observed at both the middle and high-frequency regions for the Mo and the Mo-Ti-Zr, suggesting the presence of two time-constants. Therefore, the EIS spectra were fitted using an equivalent circuit diagram (EEC) of *R*_s_(*Q*_p_(*R*_p_(*Q*_ct_*R*_ct_))). Where *R*_s_ represents the resistance of Hank's solution; *Q*_p_ and *R*_p_ correspond to the capacitance and resistance of the surface oxide layer; and *Q*_ct_ and *R*_ct_ are associated with the double layer capacitance and the resistance linked to the interfacial charge transfer reaction. The fitting results showed a higher *R*_ct_ value for the Mo-Ti-Zr than for the Mo, corroborating improved corrosion resistance (Table S1, Supplementary Materials).

In a long-term course of immersion degradation, the OCP curves of the pure Mo and the Mo-Ti-Zr after 7, 14, 28, and 56 days in Hank's solution indicated that the potential decreased initially before stabilizing, and then remained relatively stable (Fig. 2B1 and Fig. 2C1). Likewise, the PDP curves of the pure Mo and the Mo-Ti-Zr alloy demonstrated similar trends (Fig. 2B2 and Fig. 2C2). As immersion time increased, the corrosion potential of both samples became more negative, and both the cathodic and anodic branches shifted to the right direction, resulting in faster corrosion current density. Notably, an obvious passivation region appeared in the anodic section for both the Mo and the Mo-Ti-Zr, and the passivation zone grew wider with the prolonged immersion time, likely due to the formation of a thicker protective oxide layer on the surface [21,30]. From the related parameters of PDP curves, including the *E*_corr_, *i*_corr_, and corrosion rate (Fig. 2D1 and Fig. 2D2), it is evident that after 56 days of immersion, the *E*_corr_ values of the two materials became more closer attributable to the similar oxides formed out-most. Although the *i*_corr_ and corrosion rate of both the TZM and the Mo increased with immersion time, their values for the TZM were lower than those for pure Mo. After 56 days of immersion, the corrosion rates for the TZM and pure Mo were 1877.14 ± 225.52 and 3425.40 ± 296.02 μm/year, respectively.

The Nyquist plots for Mo and TZM showed that the impedance of both materials decreased with the prolonged immersion time (Fig. 2B3 and Fig. 2C3). The Nyquist curves for Mo after 7, 14, and 28 days of immersion exhibited a capacitive loop in the high-frequency region and a linear tail in the low-frequency zone (approximately 45°), with only a slight linear tail observed after 56 days of immersion (Fig. 2B3). In contrast, the Nyquist curves of the TZM displayed a pronounced linear tail throughout the entire immersion period (Fig. 2C3), the appearance of which was closely related to the morphology of surface corrosion products [18,30]. Bode-impedance plots for Mo and the Mo-Ti-Zr showed that the Z value decreased with increasing immersion time (Fig. 2B4 and Fig. 2C4). In the Bode-phase plot, two distinct peaks were observed in the low-frequency region (around 10^−2^-10° Hz, associated with interfacial charge transfer) and the middle-frequency region (around 10^3^–10^4^ Hz, related to the surface oxide layer characteristics), indicating the presence of two time-constant. Therefore, the EIS spectra were fitted using an equivalent electrical circuit of *R*_s_(*Q*_p_(*R*_p_(*Q*_ct_(*R*_ct_(*Z*_w_))))), where *R*_s_, *Q*_p_, *R*_p_, *Q*_ct_, and *R*_ct_ are as described earlier. *Z*_w_ refers to the Warburg impedance, which is associated with the oxygen diffusion process. The fitting results indicated that Mo-Ti-Zr displayed higher *R*_ct_ values at each time point compared to Mo (Table S2, Supplementary Materials), suggesting relatively better controlled corrosion.

The variation of pH value of Hank's solution with immersion of the materials over time was also assessed, with both the Mo-Ti-Zr and Mo demonstrating a similar trend: an initial rise in pH (peaking around 9), followed by a gradual decrease, and stabilizing at ∼pH 7 (Fig. 2E). The decrease in pH is likely related to the continuous dissolution of corrosion products, which generates H^+^ ions [21]. The corrosion rates, calculated using the weight-loss method (Fig. 2F), indicated that Mo-Ti-Zr has a lower corrosion rate (37.4 ± 4.7 μm/year) than Mo (52.1 ± 6.1 μm/year). Of note, the corrosion rates obtained from the weight-loss method differ by an order of magnitude from those calculated using the PDP curves, a phenomenon similarly reported in other degradable metals [33,34].

**Fig. 3:**
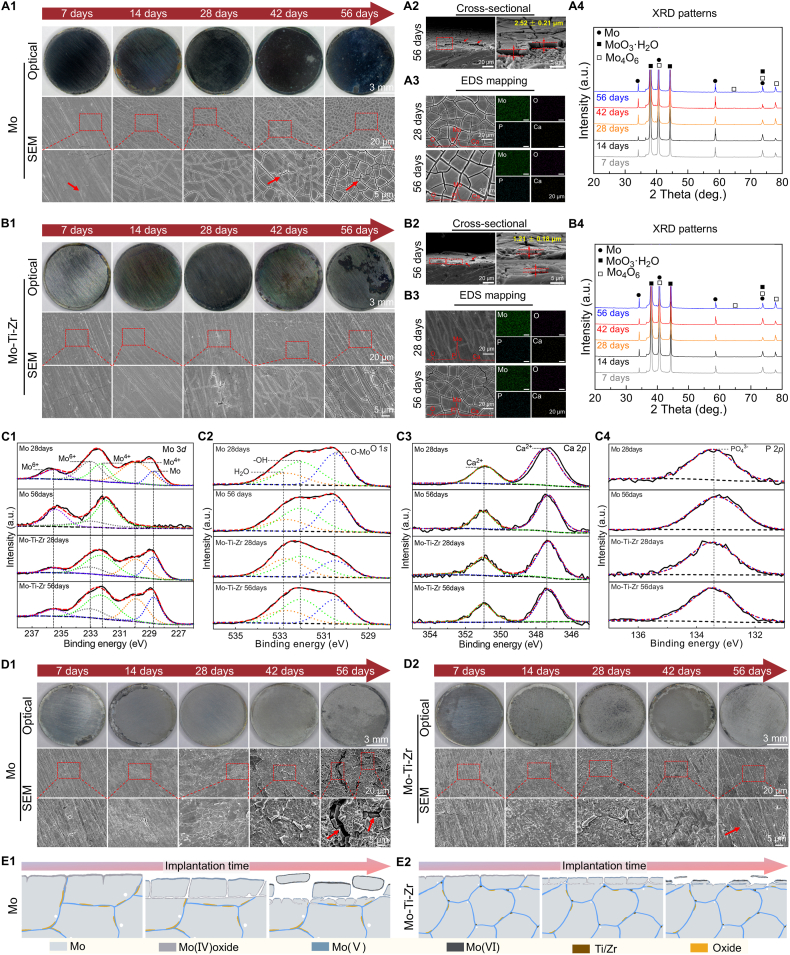
*In vitro* long-term immersion test of Mo and Mo-Ti-Zr immersed in Hank's solution at semi-static condition at 37 °C for 7, 14, 28, 42, and 56 days. (A1) Representative optical and SEM morphology of pure Mo. (A2) Cross-sectional SEM images of Mo after 56 days soaking. (A3) EDS mapping embedded with EDS curves of Mo after 28 and 56 days of immersion. (A4) XRD patterns of Mo. (B1) Representative optical and SEM morphology of Mo-Ti-Zr. (B2) Cross-sectional SEM images of Mo-Ti-Zr after 56 days soaking. (B3) EDS mapping and curves of Mo-Ti-Zr after 28 and 56 days of immersion. (B4) XRD patterns of Mo-Ti-Zr. (C1–C4) High-resolution XPS of Mo 3*d*, O 1*s*, Ca 2*p*, and P 2*p* of Mo and Mo-Ti-Zr after 28 and 56 days of soaking, respectively. (D1 and D2) Representative optical images and SEM morphology of Mo and Mo-Ti-Zr after removing surface corrosion products, respectively. (E1 and E2) Potential corrosion mechanism of Mo and Mo-Ti-Zr with the implantation time.

The surface morphology and compositional characterization of the corrosion products of Mo and Mo-Ti-Zr after 7, 14, 28, 42, and 56 days of immersion in Hank's solution were observed (Fig. 3). The color of the corrosion products on Mo changed significantly during immersion (Fig. 3A1). In the early immersion stages (7 and 14 days), the surface appeared dark gray, followed by a transition to dark black at 28 and 42 days, and eventually turning blue after 56 days of immersion. In contrast, the macroscopic optical images of Mo-Ti-Zr (Fig. 3B1) showed that the sample maintained a metallic luster at the beginning of immersion (7 days), with a slight green appearance at the edges by 14 days, gray at 28 days, yellow-green mixed areas at 42 days, and aggregated black areas on the surface after 56 days of immersion, reflecting the dynamic changes in surface corrosion products of Mo and Mo-Ti-Zr during immersion. Moreover, mechanical polishing marks were still visible on the surface of Mo samples during the first 28 days of immersion, while surface scratches on Mo-Ti-Zr samples were observable throughout the entire immersion process, likely due to differences in the thickness of the corrosion products. From the SEM images (Fig. 3A1), the corrosion products on Mo exhibited a regular flaky crystal morphology with gullies or gaps between the crystals. The size of the flaky crystals decreased with prolonged immersion time. After 42 and 56 days of immersion, defects caused by the detachment of flaky crystals in the gullies were observed (indicated by red arrows). In contrast, the morphology of the corrosion products on Mo-Ti-Zr was similar to that of Mo but more uniform (Fig. 3B1). The thickness of the corrosion products on the Mo surface was 2.52 ± 0.21 μm, while that in the corrosion product layer on Mo-Ti-Zr was half as thick, at 1.21 ± 0.18 μm (Fig. 3A2 and Fig. 3B2). Additionally, lifting or peeling of corrosion products indicates loosely adhesion between the corrosion products and the substrate (Fig. 3A2 and Fig. 3B2), as reported in previous studies [21,30]. EDS mapping (Fig. 3A3 and Fig. 3B3) and semi-quantitative data (Table S3, Supplementary Materials) of the surface corrosion products after 28 and 56 days of immersion showed that the corrosion products on both Mo and Mo-Ti-Zr were primarily composed of Mo, O, and C, with small amounts of P and Ca. With increasing immersion time, the content of Mo and C elements decreased, while the content of O, P, and Ca increased; no Ti or Zr element was detected on the Mo-Ti-Zr surface, likely because the contents of these elements were below the detection limit of the instrument. XRD (Fig. 3A4 and Fig. 3B4) further confirmed the phase composition and structure of the surface corrosion products, showing that the main components were MoO_3_ and Mo_4_O_6_.

X-ray photoelectron spectroscopy (XPS) data of Mo and Mo-Ti-Zr after 28 and 56 days of immersion showed characteristic peaks of Mo 3*d*, O 1*s*, Ca 2*p*, and P 2*p* (Fig. S1A, Supplementary Materials). Additionally, the content of Mo and C decreased with prolonged immersion, while those of O, Ca, and P increased, consistent with the semi-quantitative results obtained by EDS (Table S4, Supplementary Materials). High-resolution spectra for Mo 3*d* (Fig. 3C1) showed peaks for Mo (ca. 228.8 eV), Mo^4+^ (ca. 229.9 and ca. 232.3 eV), and Mo^6+^ (ca. 233.1 and 235.6 eV) after 28 days of immersion. After 56 days, the Mo peak was no longer visible in pure Mo sample, leaving only Mo^4+^ and Mo^6+^ peaks, indicating dynamic changes in surface corrosion products. In contrast, the Mo-Ti-Zr group exhibited Mo, Mo^4+^, and Mo^6+^ peaks at both immersion times, with a slight increase in the Mo^6+^ peak over time, suggesting an oxidation progression from lower to higher valence states [21]. The high-resolution spectra of O 1*s*, Ca 2*p*, and P 2*p* for Mo-Ti-Zr and Mo showed: O-Mo, -OH, and H_2_O peaks were observed in the O 1*s* spectrum (Fig. 3C2), a Ca^2+^ peak in the Ca 2*p* spectra (Fig. 3C3), and a PO_4_^3−^ peak in the P curve (Fig. 3C4). Additionally, a high-resolution Ti 2*p* signal was detected in the Mo-Ti-Zr samples (Fig. S1B, Supplementary Materials), while no distinct Zr 3*d* signal was observed (Fig. S1C, Supplementary Materials).

Following the removal of surface corrosion products, both Mo and Mo-Ti-Zr had a metallic matrix, with surface scratches from mechanical polishing gradually becoming shallower over time (Fig. 3D1 and Fig. 3D2). Small pores and gaps on the Mo surface were observed, with their size increasing with prolonged immersion time; prolonged immersion leads to the dissolution of crystals around these gaps, resulting in more prominent cracks. In contrast, the Mo-Ti-Zr surface remained relatively smooth, with no large cracks observed, albeit with the appearance of some small pits after 56 days of immersion (Fig. 3E1 and Fig. 3E2). This indicates a more uniform corrosion pattern for Mo-Ti-Zr compared to Mo. The potential corrosion mechanisms of Mo and Mo-Ti-Zr with the immersed time will be discussed in detailed in later sections.

### 2.3 *In vitro* angiogenic activity of Mo-Ti-Zr compared with pure Mo

**Fig. 4:**
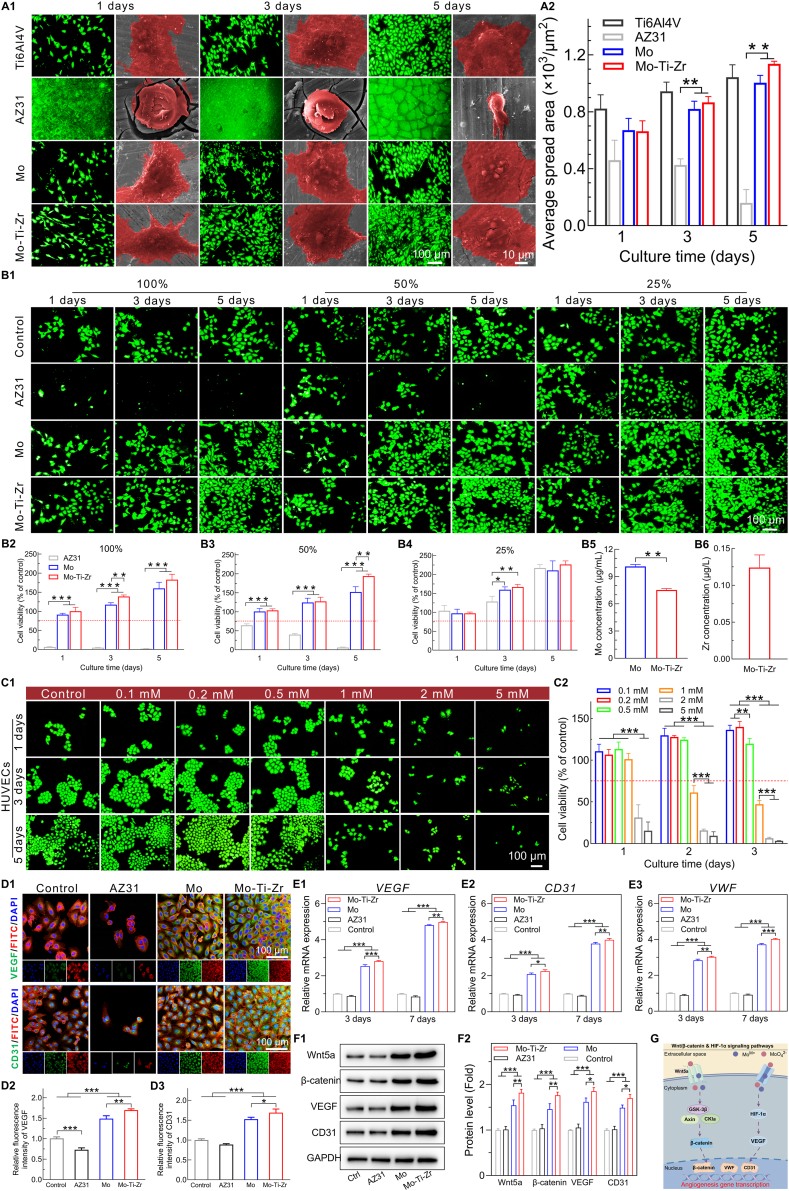
*In vitro* cytocompatibility and angiogenic activity of Mo and Mo-Ti-Zr compared to AZ31 and Ti6Al4V groups. (A1) Representative rhodamine staining fluorescence images and SEM morphology of HUVECs directly cultured on the surfaces of samples. (A2) Quantification of average spread area of HUVECs. (B1) Representative rhodamine staining fluorescence microscopy images of HUVECs cultured with 100 %, 50 %, and 25 % extracts. (B2–B4) Cell counting kit-8 (CCK-8) assay of HUVECs incubated with 100 %, 50 %, and 25 % extracts, respectively. (B5 and B6) Mo and Zr ions in 100 % extraction (DMEM/F12), respectively. (C1 and C2) Representative rhodamine staining fluorescence microscopy images and CCK-8 assay of HUVECs cultured with different concentrations of MoCl_5_ (0.1, 0.2, 0.5, 1, 2, and 5 mM) for 1, 3, and 5 days, respectively. (D1) Immunofluorescence staining of HUVCEs for VEGF and CD31 after incubation with 50 % extracts for 3 days. VEGF and CD31 were stained green, F‐actin was stained red and the nuclei were stained blue. (D2 and D3) Semi‐quantitative analysis of fluorescence intensity of VEGF and CD31, respectively. (E1–E3) *VEGF*, *CD31*, and VWF gene expression in HUVECs, respectively. (F1 and F2) Western blotting and quantification of VEGF, CD31, β-catenin protein, and Wnt5a expression in HUVECs, respectively. (G) Potential pro-angiogenesis mechanism of Mo-Ti-Zr. Data represents mean ± standard deviation (*n* = 4). ∗*p* < 0.05, ∗∗*p* < 0.01, ∗∗∗*p* < 0.001 compared between group. Data represents mean ± standard deviation (*n* = 4). ∗*p* < 0.05, ∗∗*p* < 0.01, ∗∗∗*p* < 0.001 compared between group. (For interpretation of the references to color in this figure legend, the reader is referred to the Web version of this article.)

The adhesion, proliferation, and angiogenic activity of human umbilical vein endothelial cells (HUVECs) were investigated by direct incubation with the samples and in cultures with sample extracts at 100 %, 50 %, and 25 % concentrations. Rhodamine fluorescence staining and SEM images of HUVECs on the surfaces of Ti6Al4V, AZ31, Mo, and Mo-Ti-Zr after 1, 3, and 5 days of culture showed only a few spherical cells on the AZ31 surface; in contrast, the cell density on Mo and Mo-Ti-Zr surfaces increased over time, with the Mo-Ti-Zr surface exhibiting a cell count comparable to Ti and higher than Mo (Fig. 4A1). HUVECs on the AZ31 surface appeared shrunk and spherical, with collapsed edges, indicating poor adhesion, while cells on the Mo-Ti-Zr and Mo surfaces display prominent pseudopodia and a well-spread morphology. Statistical data on cell spreading area showed a significant increase in average cell spreading area on Mo-Ti-Zr and Mo surfaces over time, far exceeding that on AZ31 and comparable to that on Ti (Fig. 4A2).

Rhodamine fluorescence staining images were obtained for HUVECs cultured in 100 %, 50 %, and 25 % extracts for 1, 3, and 5 days (Fig. 4B1). In the 100 % extract, almost no cells were observed on the AZ31, indicating high cytotoxicity, whereas cell density on Mo and Mo-Ti-Zr groups increased over time, comparable to that of the control group (cultured with normal cell medium). In the 50 % extract, the Mo and Mo-Ti-Zr groups exhibited significantly higher cell counts than both the control and AZ31 groups. In the 25 % extract, the Mo and Mo-Ti-Zr groups also showed a markedly higher cell count than the control group, indicating a stimulatory effect on HUVEC proliferation. Quantitative results demonstrated that, in the 100 % extract, the cell viability in Mo and Mo-Ti-Zr was significantly higher than that in AZ31, with viability exceeding 75 % (indicated by the red dashed line) (Fig. 4B2), suggesting no cytotoxicity according to the standard of ISO 10993-12 [35]. Additionally, the cell viability on Mo and Mo-Ti-Zr increased over time, particularly after 5 days, where it significantly surpassed the control group (100 %), indicating a promotive effect on HUVEC proliferation. In the 50 % extract, cell viability showed a trend similar to that observed in the 100 % extract, with Mo-Ti-Zr showing higher viability than Mo after 5 days (Fig. 4B3). In the 25 % extract, cell viability of Mo and Mo-Ti-Zr was comparable to AZ31, and significantly higher than the control group at 3 and 5 days (Fig. 4B4). These results indicate that Mo and Mo-Ti-Zr are non-toxic to HUVECs, and diluted extracts promote cell proliferation and adhesion. The concentration of Mo in the 100 % extract, showing that Mo concentration in the Mo-Ti-Zr group (ca.10 μg/mL; ca. 0.10 mM) was lower than in the pure Mo group (ca. 7.10 μg/mL; ca. 0.07 mM) (Fig. 4B5), with Zr concentration in the 100 % extract being approximately 0.13 μg/mL (Fig. 4B6).

To further investigate the effects of Mo ion concentration on HUVEC activity, cells were cultured for 1, 2, and 3 days in media containing 0.1, 0.2, 0.5, 1, 2, and 5 mM Mo ions (Mo^5+^). At concentrations of 0.1, 0.2, and 0.5 mM (Fig. 4C1), the number of HUVECs increased over time, with cells maintaining normal morphology. Notably, cell counts at 2 and 3 days were significantly higher than in the control group (cultured with normal cell medium). At 1 mM, cell numbers decreased with extended culture time, and at 2 and 5 mM, cells exhibited a shrunken, spherical morphology, with only a minimal number of viable cells observed. Mo ion concentrations of 0.1, 0.2, and 0.5 mM had no toxic effect on HUVECs (viability above the 75 % threshold marked by the red dashed line) and promoted cell proliferation after 2 and 3 days of culture (viability greater than 100 %) (Fig. 4C2). In contrast, Mo ions at 1 mM showed no cytotoxicity only after 1 day of incubation but significant cytotoxicity after 2 and 3 days of culture (Fig. 4C2). Mo ion concentrations of 2 and 5 mM exhibited evident cytotoxicity (Fig. 4C2). Thus, the response of HUVECs to Mo ions is dependent on both concentration and time, a phenomenon common to other biodegradable metals [36,37]. Similarly, the effects of Zr ion concentration on HUVECs were examined (Supplementary Fig. S2). After 3 days of culture, Zr ions at 5–50 μM progressively enhanced cell viability and up-regulated the angiogenic markers CD31 and VEGF. At 100 μM, the pro-angiogenic effect was still evident but less pronounced than at 50 μM. In contrast, higher concentrations (≥500 μM) induced marked cytotoxicity, with decreased viability and downregulated gene expression compared with the control. These findings indicate that Zr ions exert a concentration-dependent effect on endothelial cell activity, with low-to-moderate concentrations (5–50 μM) being most favorable, while excessive exposure is inhibitory.

The expression of vascular endothelial growth factor (VEGF) and cluster of differentiation 31 (CD31) factors in HUVECs cultured for 5 days in 50 % extracts of Mo and Mo-Ti-Zr was assessed using laser confocal microscopy. AZ31 led to a low VEGF and CD31 expression (Fig. 4D1). In contrast, HUVECs in the Mo-Ti-Zr and Mo groups displayed significantly higher expression levels of VEGF and CD31. Quantitative results (Fig. 4D2 and Fig. 4D3) further confirmed these findings, with VEGF and CD31 expression notably higher in the Mo-Ti-Zr group compared to pure Mo. The expression of angiogenesis-related genes in HUVECs, including VEGF, CD31, and von Willebrand factor (VWF), was determined using real-time quantitative polymerase chain reaction (RT-qPCR) (Fig. 4E1-E3). Mo and Mo-Ti-Zr showed higher VEGF, CD31, and VWF expression levels than AZ31 and control groups, with Mo-Ti-Zr exhibiting the highest level. The protein expression of VEGF, CD31, and Wnt/β-catenin signaling pathway-related proteins (Wnt5a and β-catenin) was validated using western blotting (WB) (Fig. 4F1 and Fig. 4F2). Both proteins were up-regulated in HUVECs exposed to Mo and Mo-Ti-Zr sample extract compared to the AZ31 and control groups, with Mo-Ti-Zr showing the highest expression level. The potential biological mechanism for Mo-Ti-Zr induced angiogenesis was proposed based on the above results (Fig. 4G). The Mo element and MoO_4_^2−^ in the extract may activate angiogenesis-related signaling pathways in HUVECs, such as the Wnt/β-catenin and hypoxia-inducible factor 1α (HIF-1α) pathways, thereby promoting the expression of angiogenesis-related proteins and facilitating angiogenesis.

### 2.4 *In vitro* performance and osteogenic mechanism of Mo-Ti-Zr compared with pure Mo

#### 2.4.1 Osteogenic activity

**Fig. 5:**
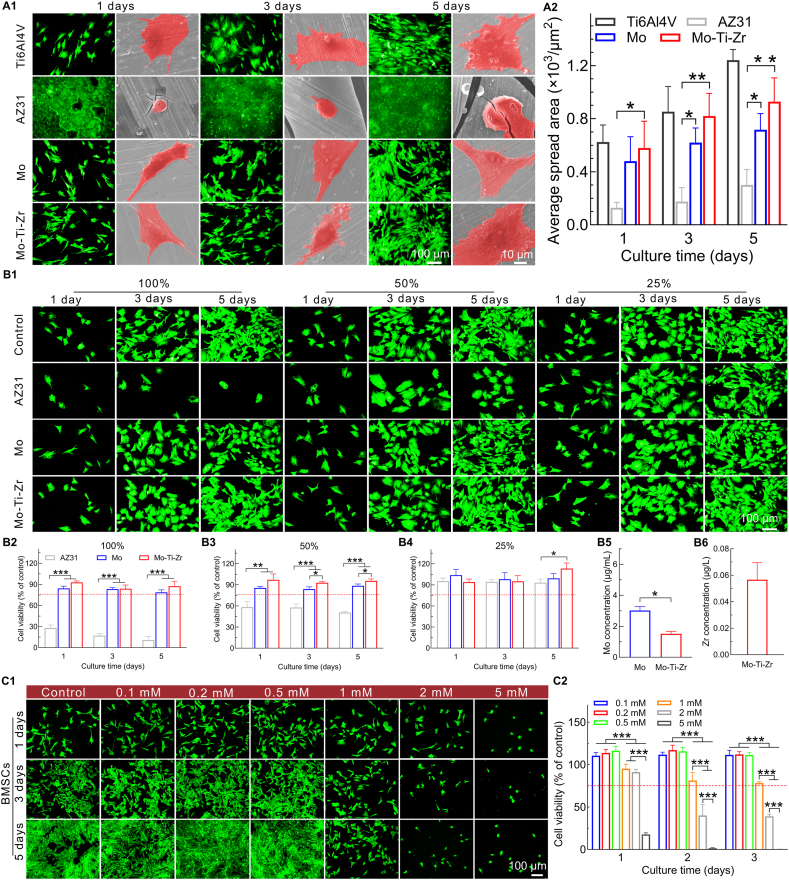
*In vitro* cytocompatibility of Mo and Mo-Ti-Zr compared to AZ31 and Ti6Al4V groups. (A1) Representative rhodamine staining fluorescence images and SEM morphology of BMSCSs directly cultured on the surfaces of samples. (A2) Quantification of average spread area of BMSCSs. (B1) Representative rhodamine staining fluorescence microscopy images of BMSCSs cultured with 100 %, 50 %, and 25 % extracts. (B2–B4) Cell counting kit-8 (CCK-8) assay of BMSCSs incubated with 100 %, 50 %, and 25 % extracts, respectively. (B5 and B6) Mo and Zr ions in 100 % extraction (αMEM), respectively. (C1 and C2) Representative rhodamine staining fluorescence microscopy images and CCK-8 assay of BMSCs cultured with different concentrations of MoCl_5_ (0.1, 0.2, 0.5, 1, 2, and 5 mM) for 1, 3, and 5 days, respectively. Data represents mean ± standard deviation (*n* = 4). ∗*p* < 0.05, ∗∗*p* < 0.01, ∗∗∗*p* < 0.001 compared between groups.

Rhodamine fluorescence staining and SEM images of bone marrow mesenchymal stem cells (BMSCs) directly cultured on the surfaces of Ti6Al4V, AZ31, Mo, and Mo-Ti-Zr showed that cells on the AZ31 surface appear spherical and sparse, while those on Mo and Mo-Ti-Zr surfaces proliferated in a manner comparable to the Ti group and exhibited a natural morphology, well-spread with abundant pseudopodia (Fig. 5A1). Cell spreading on Mo and Mo-Ti-Zr surfaces was significantly greater than on AZ31 (Fig. 5A2). In the 100 % extract, cell density on Mo-Ti-Zr and Mo surfaces increased with time and was higher than in both the control and AZ31 groups (Fig. 5B1). At 50 % and 25 % extract concentrations, cell density was even higher, indicating that diluted extracts are more conducive to cell proliferation. Quantitative cell viability data show that, in the 100 % extract (Fig. 5B2), cell viability in the Mo and Mo-Ti-Zr groups was significantly higher than in the AZ31 group at all time points, with Mo-Ti-Zr consistently exhibiting the highest viability. This trend remained evident at 50 % concentration (Fig. 5B3), where Mo-Ti-Zr and Mo maintained a high viability, while AZ31 showed a significant reduction in cell viability. At 25 % concentration (Fig. 5B4), cell viability in the Mo and Mo-Ti-Zr groups was stable and comparable at all time points. Thus, Mo-Ti-Zr possesses excellent biocompatibility and supports BMSCs proliferation across various concentrations and culture times. The concentration of Mo in the 100 % extract (Fig. 5B5 and Fig. 5B6), showing a similar trend to that in HUVECs, but the concentration is relatively low. The Mo concentration is ca. 3 μg/mL (ca. 0.03 mM) for Mo, and ca. 1.5 μg/mL (ca. 0.02 mM) for Mo-Ti-Zr.

In fluorescence-stained images (Fig. 5C1) and cell viability assay (Fig. 5C2), a clear trend was observed for BMSCs cultured with different Mo ion (Mo^5+^) concentrations (0.1, 0.2, 0.5, 1, 2, and 5 mM) over 1, 3, and 5 days. At lower concentrations (0.1, 0.2, and 0.5 mM), cell density and viability increased with culture time, indicating that these concentrations are favorable for BMSCs proliferation. However, at higher concentrations (1, 2, and 5 mM), cell density and viability decreased significantly, with 2 and 5 mM concentrations displaying clear cytotoxic effects, as evidenced by reduced cell counts and poor morphology. This pattern was also reflected in the cell viability, where cell viability remained high for lower Mo ion concentrations but decreased sharply for higher concentrations. Thus, low concentrations of Mo ions support cell growth, while high concentrations inhibit it. Previous studies have shown that BMSCs also display a concentration-dependent response to MoO_3_, one of the corrosion products of Mo [22,23]. Similarly, when BMSCs were exposed to Zr ions (Supplementary Fig. S3), a graded pro-osteogenic response was observed: cell viability and the expression of Runx2 and BMP-2 increased progressively from 5 to 100 μM, whereas 500 μM caused obvious cytotoxicity (and 1000 μM led to marked cell death).

**Fig. 6:**
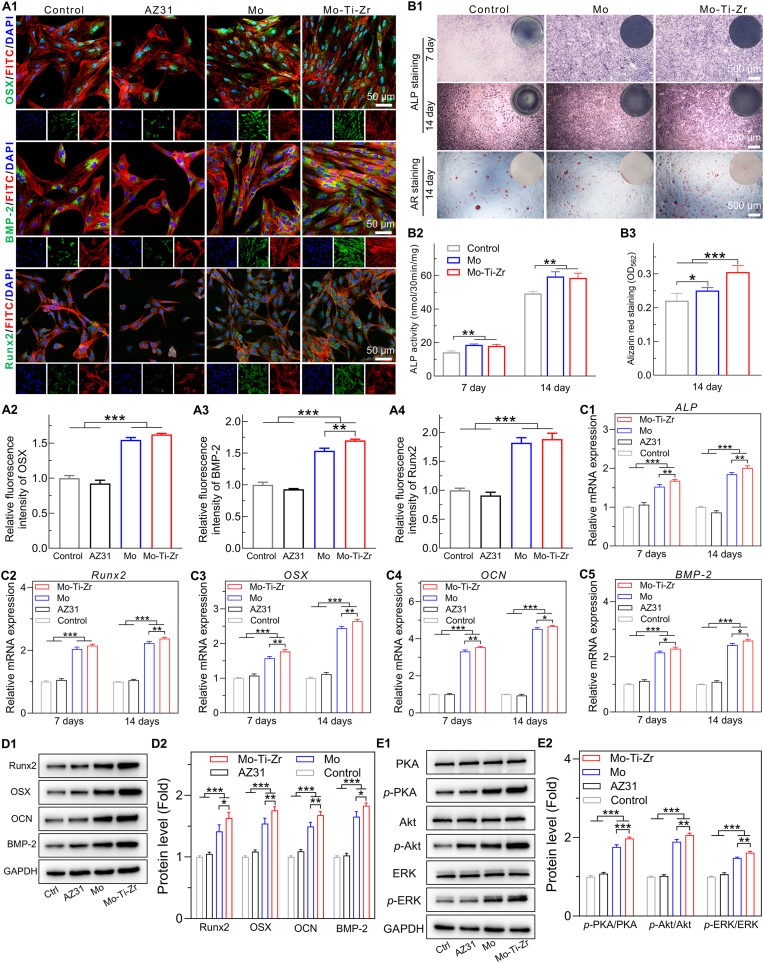
*In vitro* osteogenic activity of Mo and Mo-Ti-Zr samples. (A1) Immunofluorescence staining of BMSCs for OSX, BMP-2, and Runx2 after incubation with 50 % extracts for 7 days. OSX, BMP-2, and Runx2 were stained green, F‐actin was stained red and the nuclei were stained blue. (A2–A4) Semi‐quantitative analysis of fluorescence intensity of OSX, BMP-2, and Runx2, respectively. (B1) Representative micrographs of alkaline phosphatase (ALP) and alizarin red (AR) staining of BMSCs after 7 and 14 days of incubation. (B2 and B3) Quantification of ALP expression and calcium nodules, respectively (*n* = 3). (C1-C5) *ALP*, *Runx2*, *OSX*, *OCN*, and *BMP-2* gene expression in BMSCs, respectively. (D1 and D2) Western blotting and quantification of Runx2, OSX, OCN, and BMP-2 proteins in BMSCs, respectively. (E1 and E2) Western blotting and quantification of p-PI3K/PI3K, p-ERK/ERK, and p-PKA/PKA protein expression in BMSCs, respectively. Data represents mean ± standard deviation (*n* = 4). ∗*p* < 0.05, ∗∗*p* < 0.01, ∗∗∗*p* < 0.001 compared between groups. (For interpretation of the references to color in this figure legend, the reader is referred to the Web version of this article.)

Immunofluorescence staining for osteogenic proteins, including osterix (OSX), bone morphogenetic protein 2 (BMP-2), and runt-related transcription factor 2 (Runx-2), was performed in BMSCs cultured in 50 % Mo and Mo-Ti-Zr extracts for 5 days (Fig. 6A1). The fluorescence intensity of OSX, BMP-2, and Runx-2 in the Mo and Mo-Ti-Zr groups was significantly higher than in the control and AZ31 groups, indicating enhanced osteogenic differentiation. Notably, the BMP-2 fluorescence intensity in the Mo-Ti-Zr group was significantly higher than in the Mo group, suggesting a more pronounced osteogenic effect. Quantitative analysis (Fig. 6A2, Fig. 6A3, and Fig. 6A4) of fluorescence intensity further supports these findings.

Alkaline phosphatase (ALP) and alizarin red (AR) staining was performed on BMSCs cultured in 50 % Mo and Mo-Ti-Zr extracts for 7 and 14 days (Fig. 6B1). Mo and Mo-Ti-Zr enhanced ALP expression in BMSCs compared to the control group. Further, on day 14, the mineralization level, as determined by AR staining, in the Mo-Ti-Zr group was significantly higher than in the control and Mo groups as confirmed by quantitative analysis (Fig. 6B2 and Fig. 6B3). The expression of osteogenesis-related genes, including ALP, Runx2, OSX, OCN, and BMP-2, was evaluated (Fig. 6C1-C5). Mo and Mo-Ti-Zr displayed higher level than AZ31 and control group, with Mo-Ti-Zr showing highest level. The protein expression levels of Runx2, OSX, OCN, and BMP-2 exhibited a trend similar to that observed in the gene expression results (Fig. 6D1 and Fig. 6D2). The expression of key proteins associated with phosphoinositide 3-kinase–Akt (PI3K–Akt), mitogen-activated protein kinase (MAPK-ERK), and cyclic adenosine monophosphate (cAMP-PKA), including Akt, phosphorylated Akt (p-Akt), ERK, phosphorylated ERK (p-ERK), PKA, and phosphorylated PKA (p-PKA), as well as total GAPDH, were evaluated (Fig. 6E1 and Fig. 6E2). Compared with control and AZ31 groups, the Mo and Mo-Ti-Zr sample extracts demonstrated a similar trend for aforementioned osteogenesis-related genes, and the latter showed the strongest signaling.

#### 2.4.2 Transcription sequencing of BMSCs

**Fig. 7:**
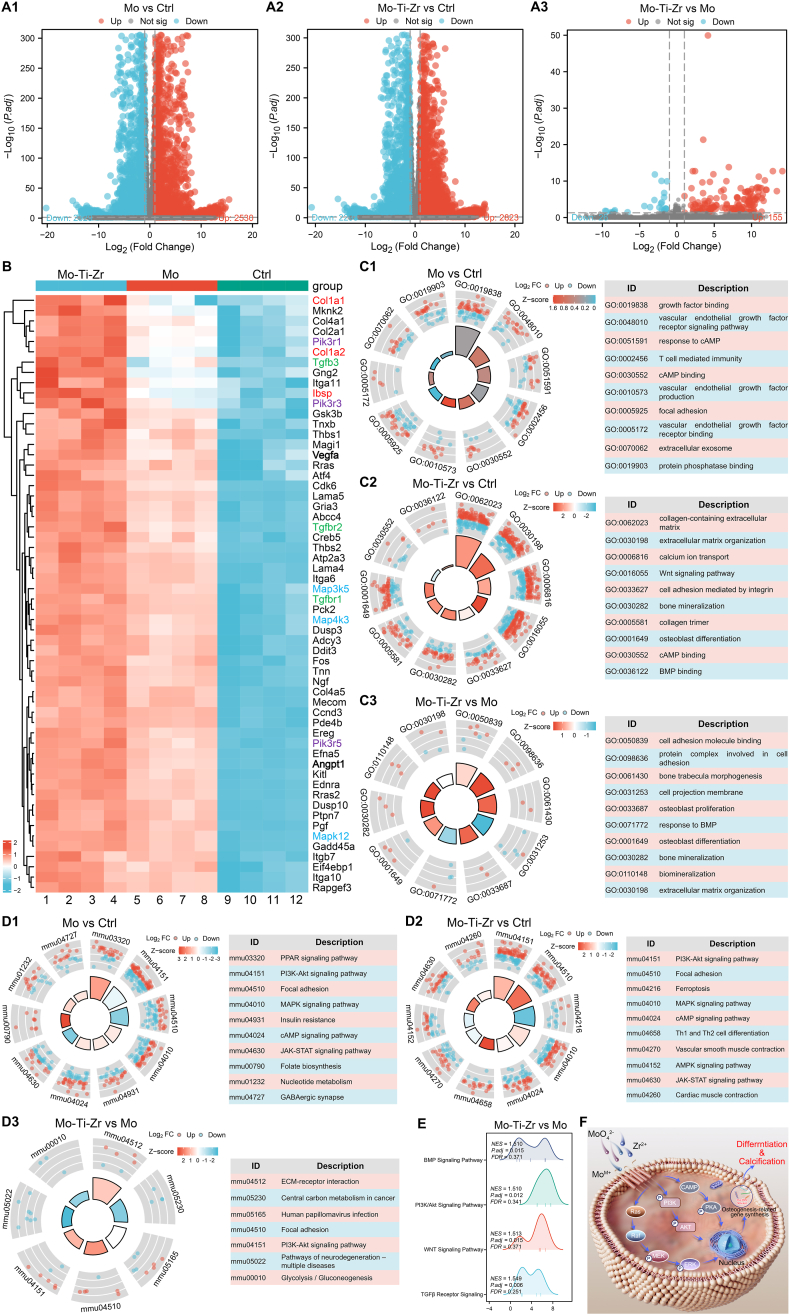
Transcriptome sequencing of BMSCs cultured with 50 % Mo and Mo-Ti-Zr extract compared with the control group. (A1–A3) Volcano plots of the differentially expressed genes (DEGs) of Mo vs. control (Ctrl), Mo-Ti-Zr vs. Ctrl, and Mo-Ti-Zr vs. Mo, with up-regulated genes in red, downregulated genes in blue, and unchanged genes in gray. (B) A heatmap of DEGs between Mo-Ti-Zr, Mo, and Ctrl group, with osteogenesis-related genes labeled in red, violet, green, and blue color, angiogenesis-related genes bolded text. (C1–C3) Gene ontology (GO) enrichment plot of Mo vs. Ctrl, Mo-Ti-Zr vs. Ctrl, and Mo-Ti-Zr vs. Mo. (D1–D3) KEGG analysis of the signaling pathways of Mo vs. control (Ctrl), Mo-Ti-Zr vs. Ctrl, and Mo-Ti-Zr vs. Mo. (E) GSEA enrichment plots of the positive regulatory signaling pathways of Mo-Ti-Zr vs. Mo. (F) Potential biological mechanism of osteogenesis of Mo-Ti-Zr. (For interpretation of the references to color in this figure legend, the reader is referred to the Web version of this article.)

To investigate the osteogenic mechanisms of Mo-Ti-Zr compared with Mo, BMSCs were incubated with extracts of the two materials, followed by transcriptomic sequencing and comparison with a control group (cell cultured with normal medium). BMSCs cultured in Mo displayed 2530 up-regulated differentially expressed genes (DEGs; marked in red) and 2329 downregulated DEGs (labeled in blue) compared to the control group (Fig. 7A1). The Mo-Ti-Zr group had 2823 up-regulated DEGs and 2293 downregulated DEGs (Fig. 7A2) compared to the control group. A further comparison between Mo-Ti-Zr and Mo revealed 155 up-regulated and 26 downregulated DEGs in the Mo-Ti-Zr group. Compared with the control and Mo groups, Mo-Ti-Zr extracts significantly up-regulated key osteogenesis-related genes (marked in red; Fig. 7B) such as collagen type Iα 1/2 chain (ColIα1, ColIα2) and integrin-binding sialoprotein (Ibsp). *ColIα1* and *ColIα2* encode the α1 and α2 chains of type I collagen (Col I), respectively, which together form Col I, a major component of bone matrix [38]. Ibsp is a primary non-collagenous protein in the bone matrix, playing a critical role in mineral nodule formation and calcium phosphate deposition [39]. Additionally, key genes in the PI3K–Akt signaling pathway (*Pik3r1*, *Pik3r3*, *Pik3r5*; marked in violet), MAPK signaling pathway (*Map3k5*, *Map4k3*, *Mapk12*; marked in blue), and transforming growth factor-β signaling pathway (TGF-β; *Tgfb3*, *Tgfbr1*, *Tgfbr2*; marked in green) were notably up-regulated in cells treated with Mo-Ti-Zr extracts, suggesting activation of these pathways. *Pik3r1*, *Pik3r3*, and *Pik3r5* encode different regulatory sub-units in the PI3K–Akt pathway, which are associated with enhanced osteogenic activity and increased bone density [40]. *Map3k5*, *Map4k3*, and *Mapk12* are genes encoding components of different MAPK pathway levels, which regulate osteoblast survival and mineralization [41]. *Tgfb3*, *Tgfbr1*, and *Tgfbr2* collaboratively function in the TGFβ pathway, linked to bone matrix protein synthesis and osteogenic activity [42]. Moreover, the angiogenesis-related genes (bold text) of *Vegfa* and *angiopoietin 1 (Angpt1*) were significantly activated, crucial for vascular formation and stabilization of new vessels [43,44]. Notably, expression levels of these genes were markedly higher in the Mo-Ti-Zr group compared to the Mo-only group, further supporting the superior osteogenic potential of Mo-Ti-Zr.

To further explore the functional significance of DEGs, gene ontology (GO) analysis was performed for cellular components (CC), molecular functions (MF), and biological processes (BP) (Fig. 7C1–C3). Compared to the control group, the Mo group was significantly enriched in CC (extracellular exosome and focal adhesion), MF (vascular endothelial growth factor receptor binding and cAMP binding), and BP (vascular endothelial growth factor receptor signaling pathway, vascular endothelial growth factor production, and response to cAMP), indicating activation of VEGF-related and cAMP-related pathways (Fig. 7C1). The Mo-Ti-Zr group was enriched in CC (collagen-containing extracellular matrix and collagen trimer), MF (BMP binding), and BP (extracellular matrix organization, Wnt signaling pathway, bone mineralization, and osteoblast differentiation) compared to the control group (Fig. 7C2), all of which are highly relevant to osteoblast mineralization. Further comparison between Mo-Ti-Zr and Mo revealed that Mo-Ti-Zr was more enriched in mineralization-related BP (bone mineralization and biomineralization) and in MF (cell adhesion molecule binding) and CC (protein complex involved in cell adhesion), indicating a greater potential for promoting osteogenic mineralization and cell adhesion.

Kyoto Encyclopedia of Genes and Genomes (KEGG) pathway enrichment analysis was conducted to further explore the molecular mechanisms through which Mo and Mo-Ti-Zr promote osteogenesis (Fig. 7D1–D3). Mo extract-treated BMSCs were enriched in MAPK and cAMP signaling pathways compared to the control group (Fig. 7D1), which regulate osteoblast proliferation, differentiation, and survival [41,45], whereas it turns to inhibit the PI3K–Akt and JAK–STAT signaling pathways (Z < 0). By comparison, the Mo-Ti-Zr activated the all the PI3K–Akt, MAPK, cAMP, and JAK–STAT pathways compared to the control group and Mo(Fig. 7D2), all of which are associated with osteogenesis [40,41,45,46]. Further, significant enrichment in ECM-receptor interaction and PI3K–Akt pathways was observed in the Mo-Ti-Zr group, supporting more the role of Mo-Ti-Zr in promoting osteoblast adhesion, spreading, and matrix mineralization (Fig. 7D3). It must be emphasized that Mo-Ti-Zr activated the PI3K–Akt pathway compared to Mo.

Gene Set Enrichment Analysis (GSEA) indicated that BMSCs treated by Mo extracts showed positive enrichment in the cAMP, MAPK, Notch, and peroxisome proliferator-activated receptor (PPAR) signaling pathways (Fig. S4A, Supplementary Materials), with normalized enrichment scores (NES) exceeding 1, indicating activation of these osteogenic pathways by Mo extracts. Compared to the control, Mo-Ti-Zr showed higher enrichment in cAMP, PI3K–Akt, MAPK, Notch, and Wnt pathways, with NES values exceeding 1.3 (Fig. S4B, Supplementary Materials), suggesting that Mo-Ti-Zr significantly activated these pathways. A further comparison between Mo-Ti-Zr and Mo (Fig. 7E) indicated higher enrichment of BMP, PI3K–Akt, Wnt, and TGFβ receptor pathways in the Mo-Ti-Zr group (NES >1.5), corroborating the heatmap, GO, and KEGG analyses described above.

Transcriptomic sequencing results demonstrated that both Mo and Mo-Ti-Zr stimulated the expression of angiogenic and osteogenic genes in BMSCs and activated multiple osteogenesis-related signaling pathways. Notably, treatment with Mo-Ti-Zr extracts showed greater enrichment in genes and pathways associated with mineralization in BMSCs, significantly activating osteogenesis-related pathways such as BMP-2, TGFβ, PI3K–Akt, and Wnt, indicating enhanced osteogenic potential (Fig. 7F).

### 2.5 Hemocompatibility

Coagulation time results for Mo and Mo-Ti-Zr were obtained (Fig. S5A, Supplementary Materials). The activated partial thromboplastin time and prothrombin time were similar across all groups (Ti6Al4V, Mo, and Mo-Ti-Zr) with no significant differences. For thrombin time, the Mo-Ti-Zr group showed a slightly shorter coagulation time compared to the Mo and Ti6Al4V groups, but fall still into a quite normal range. In terms of hemolysis rate (Fig. S5B, Supplementary Materials), both Mo and Mo-Ti-Zr exhibited good performance, with Mo-Ti-Zr showing superior results of lower value over Mo.

### 2.6 *In vivo* performance of femur fracture healing

**Fig. 8:**
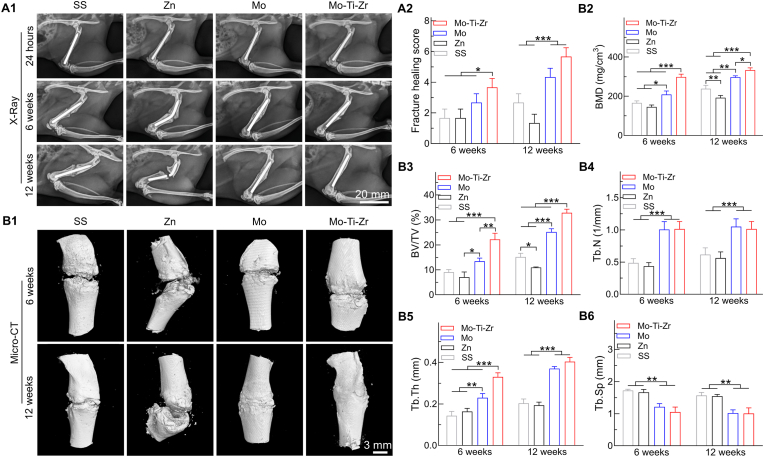
Implantation of Mo-Ti-Zr IMN compared with Mo, Zn, and SS IMNs in the rat femur shaft fracture model for 6 and 12 weeks. (A1) Representative radiographs scanning images of rat femurs containing IMNs post-surgery of 6 and 12 weeks. (A2) Fracture healing score. (B1) Representative micro-CT 3D reconstruction images of rat femur. (B2 and B3) Bone mineral density (BMD) and bone volume fraction (BV/TV), respectively. (B4–B6) Trabecular number (Tb. N), trabecular thickness (Tb. Th), and trabecular spacing (Tb. Sp). Data represents mean ± standard deviation (*n* = 6). ∗*p* < 0.05, ∗∗*p* < 0.01, ∗∗∗*p* < 0.001, compared between groups.

Ther Mo-Ti-Zr-based IMNs were utilized to evaluate their osteo-promotive ability in a rat femoral fracture model as compared with the pure Mo, Zn as well as SS counterparts. The IMNs had initially a diameter of 1.5 ± 0.05 mm and a length of 25 ± 0.1 mm (Fig. S6, Supplementary Materials). X-rays taken 24 h after surgery revealed a clear fracture line with good alignment at the fracture ends (Fig. 8A1), confirming successful IMN implantation. In the SS group, fracture healing appeared delayed, with some samples remaining in the callus mineralization stage at 12 weeks post-surgery, which is in line with previous observations [32]. At 6 and 12 weeks post-surgery, the Zn-based IMN experienced complete nail breakage, consistent with our previous studies [33,34], while the Mo and Mo-Ti-Zr groups significantly promoted fracture healing. Notably, the Mo-Ti-Zr IMN led to more enhanced callus formation and improved bone union. Fracture healing scores further support these findings (Fig. 8A2), with Mo and Mo-Ti-Zr scoring higher than the SS and Zn, and Mo-Ti-Zr achieving the highest fracture healing scores. Micro-CT images (Fig. 8B1) mirror the X-ray findings, showing that Mo and Mo-Ti-Zr outperform SS and Zn in promoting bone healing. By 12 weeks, the Mo-Ti-Zr implant led to near-complete fracture repair. Bone mineral density (BMD) was consistently higher in the Mo-Ti-Zr group at all time points, particularly at 12 weeks, where it significantly surpassed that of Mo, Zn, and SS (Fig. 8B2). The Mo-Ti-Zr group consistently exhibited the highest bone volume fraction (BV/TV; Fig. 8B3), indicating substantial new bone formation. The highest trabecular number (Tb.N; Fig. 8B4) in Mo-Ti-Zr indicates a denser trabecular structure, while trabecular thickness (Tb.Th; Fig. 8B5) increased significantly over time, indicating stronger bone architecture. Additionally, the Mo-Ti-Zr group showed lower trabecular separation (Tb.Sp; Fig. 8B6), reflecting better trabecular network connectivity, a key indicator of structural integrity. Note the Mo group performed better than SS and Zn in most parameters but was still inferior to Mo-Ti-Zr in fracture healing scores, BMD, and microstructural metrics, especially at 12 weeks. Collectively, these results highlight the clear advantage of Mo-Ti-Zr in promoting bone repair and enhancing bone quality.

**Fig. 9:**
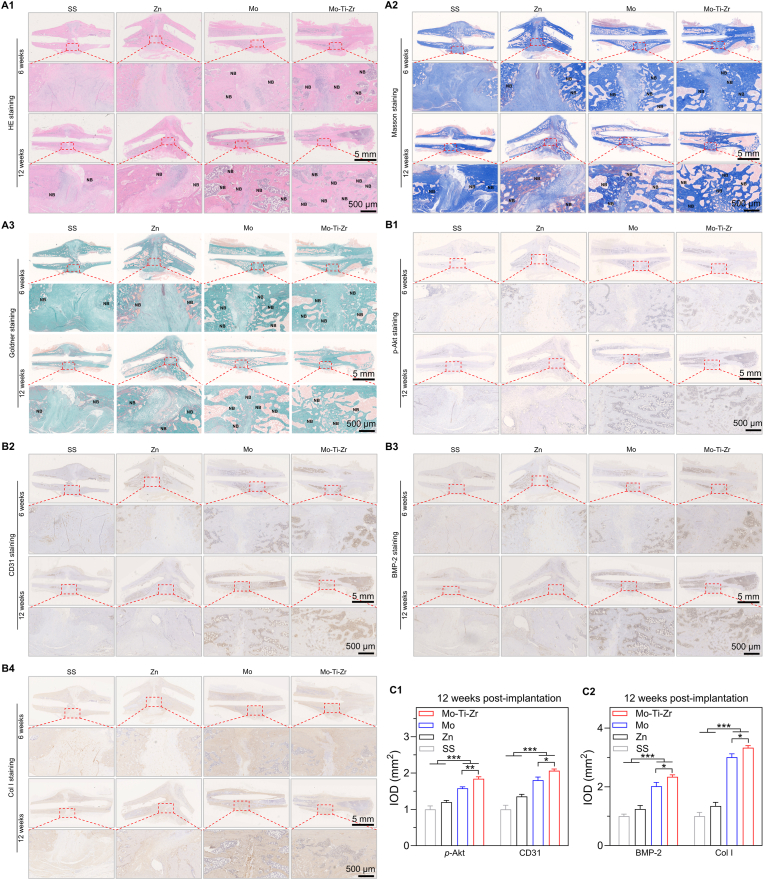
Representative staining images of rat femoral tissue at fracture site after 6 and 12 weeks implantation of intramedullary nails (IMNs). (A1–A3) H&E, Masson's trichrome, and Goldner's staining (NB indicates the newly formed bone after surgery). (B1–B4) Immunohistochemical staining of proteins related to the PI3K–Akt signaling pathway (phosphorylated Akt; p-Akt), angiogenesis (CD31), and osteogenesis (BMP-2 and ColI). (C1-C2) Quantification of p-Akt, CD31, BMP-2, and ColI after 12 weeks of implantation. Data represents mean ± standard deviation (*n* = 6). ∗*p* < 0.05, ∗∗*p* < 0.01, ∗∗∗*p* < 0.001, compared between groups.

After 6 and 12 weeks post-surgery, bone tissues at the fracture site were subjected to hematoxylin and eosin (H&E), Masson's trichome, and Goldner's staining to assess fracture healing. H&E staining (Fig. 9A1) indicated that, at 6 weeks post-surgery, the SS group still exhibited a prominent fracture line, surrounded by extensive fibrous tissue. The Zn group showed abnormal fracture healing due to the fracture, with significant fibrous tissue formation. In contrast, the Mo and Mo-Ti-Zr groups displayed a significantly smaller fracture line and more newly formed bone around the fracture site, with higher levels of bone integration. After 12 weeks, the SS group still had a visible fracture line, and the pure Zn group showed curved healing at the fracture site. However, the Mo and Mo-Ti-Zr groups exhibited more new bone formation (denoted by black NB). The collagen near the fracture site transformed into new bone trabeculae, showing a purple honeycomb-like structure, indicating good healing. Particularly, the Mo-Ti-Zr group showed almost complete healing at 12 weeks. In Masson staining (Fig. 9A2), the fracture ends in the SS and pure Zn groups were connected by abundant collagen fibers, while the Mo and Mo-Ti-Zr groups showed more collagen (stained deep blue) at both 6 and 12 weeks. Goldner staining followed a similar trend to H&E and Masson staining (Fig. 9A3), demonstrating that the Mo and Mo-Ti-Zr groups led to faster fracture healing and new bone formation (green staining) compared to the SS and pure Zn groups, with the Mo-Ti-Zr group showing the most prominent healing.

The expression of PI3K–Akt signaling pathway-related proteins (phosphorylated Akt), angiogenesis-related proteins (CD31), and osteogenesis-specific proteins (BMP-2 and ColI) was assessed. Immunohistochemical staining and corresponding integrated optical density (IOD) values were obtained (Fig. 9B1-B4 and Fig. 9C1-C2). p-Akt, a downstream protein of the PI3K–Akt signaling pathway, is involved in bone tissue growth and formation [40]. Compared to the SS, Zn, and Mo groups, Mo-Ti-Zr presented the highest expression of p-Akt, further confirming the activation of the PI3K–Akt signaling pathway. CD31, a key marker for angiogenesis [47], was expressed significantly more in the Mo and Mo-Ti-Zr groups than in the SS and Zn groups, with Mo-Ti-Zr showing the highest expression. BMP-2, an important factor for bone formation, and ColI, a structural component of new bone tissue [38], showed greater expression in the Mo and Mo-Ti-Zr groups than in the other groups, especially in the Mo-Ti-Zr group, which had the highest BMP-2 expression. These staining results suggested that the Mo-Ti-Zr-based IMN outperforms SS, Zn, and Mo in promoting bone regeneration, creating a more favorable environment for fracture healing.

**Fig. 10:**
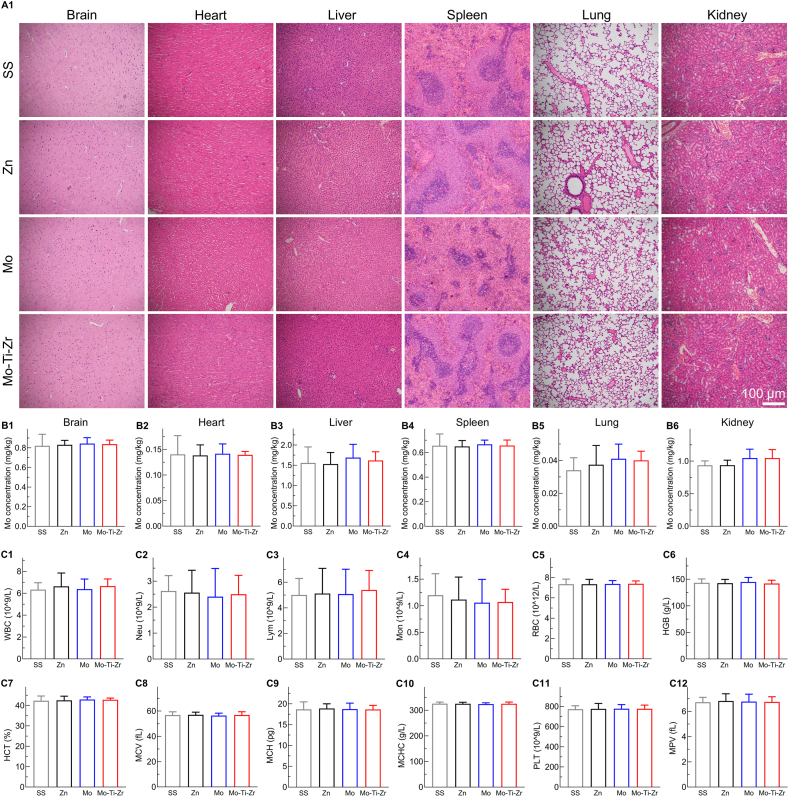
Biosafety evaluation of Mo and Mo-Ti-Zr intramedullary nails (IMNs) compared with pure Zn and SS IMNs. (A1) H&E staining of brain, heart, liver, spleen, lung, and kidney after 12 weeks of implantation in rats. (B1–B6) Mo concentration in the main organs of rats, respectively. (C1–C12) Hematological analysis of various blood parameters in response to Mo and Mo-Ti-Zr compared to Zn and SS IMNs after 12 weeks of implantation in rats, including white blood cell count (WBC), neutrophils (Neu), lymphocytes (Lym), monocytes (Mon), red blood cell count (RBC), hemoglobin (HGB), hematocrit (HCT), mean corpuscular volume (MCV), mean corpuscular hemoglobin (MCH), mean corpuscular hemoglobin concentration (MCHC), platelet count (PLT), and mean platelet volume (MPV). Data represents mean ± standard deviation (*n* = 6). (For interpretation of the references to color in this figure legend, the reader is referred to the Web version of this article.)

The H&E staining results after 12 weeks of implantation of Mo and Mo-Ti-Zr IMNs (Fig. 10A1) showed no signs of obstruction, histopathological changes, or accumulation of corrosion products in the major organs, including the brain, heart, liver, spleen, lung, and kidney. Organs exhibited normal tissue morphology without edema, inflammatory cell infiltration, or other pathological damage. Specifically, neuronal cell morphology appeared intact, with clear nuclei and distinct boundaries between white and gray matter; cardiac muscle cells displayed typical striations; hepatic cells maintained a regular polygonal shape and normal structure; spleen architecture was preserved, with well-defined white and red pulp regions; lung tissue exhibited clear alveolar structures without wall thickening; and renal tissue demonstrated well-defined glomeruli and uniform staining of tubular cells. Mo ions concentrations in major organs (Fig. 10B1–B6) showed no significant differences between the Mo-Ti-Zr, Mo, and SS groups, all remaining within a stable range. In contrast, the concentrations of Mo and Zr detected in peri-fracture tissues (Supplementary Fig. S7) were relatively higher than in normal tissues, indicating localized ion accumulation at the implant–bone interface, which is expected during the degradation process. Routine blood tests, including measurements of white blood cells (WBC), neutrophils (Neu), lymphocytes (Lym), monocytes (Mon), red blood cells (RBC), hemoglobin (HGB), hematocrit (HCT), mean corpuscular volume (MCV), mean corpuscular hemoglobin (MCH), mean corpuscular hemoglobin concentration (MCHC), platelet count (PLT), and mean platelet volume (MPV), revealed no statistically significant differences among the groups, indicating that the Mo-Ti-Zr-based IMNs exerted no adverse effects on blood parameters (Fig. 10C1–C12) and the Mo counterpart behave approximately on this aspect. Overall, these findings suggested that the Mo-Ti-Zr IMNs possess high biocompatibility and do not interfere with animal metabolism.

### 2.7 *In vivo* implant degradation behavior

**Fig. 11:**
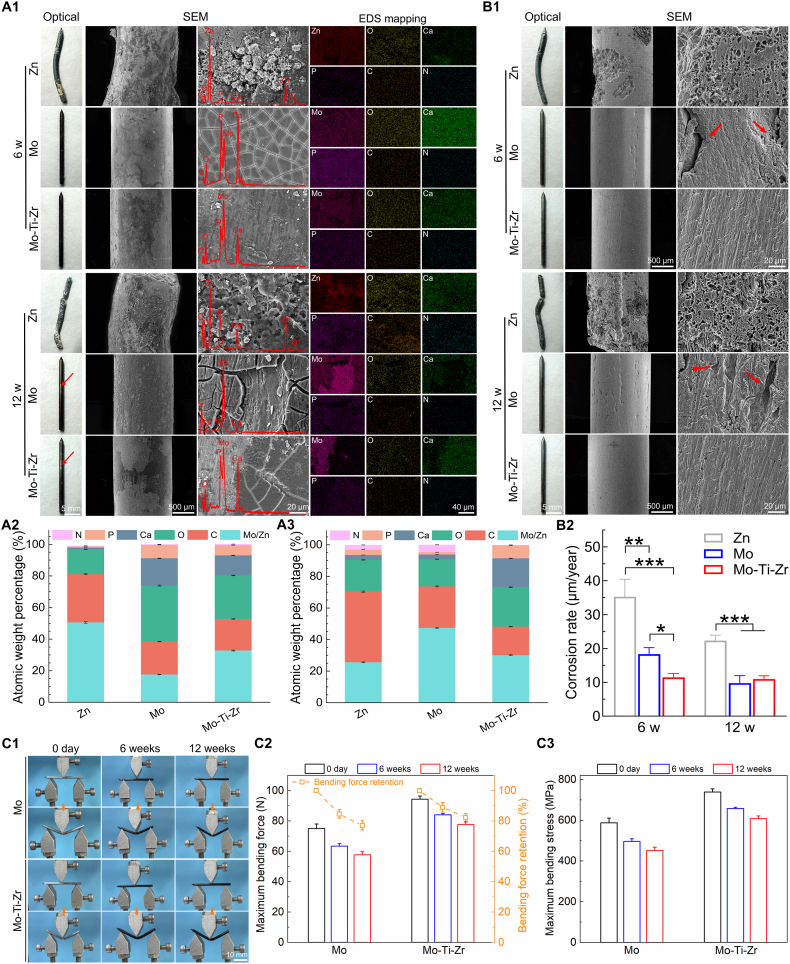
*In vivo* corrosion behavior of Mo-Ti-Zr and pure Mo intramedullary nails (IMNs) compared with bare Zn-based IMNs. (A1) Representative optical images and SEM morphology embedded with EDS mapping of Mo-Ti-Zr, Mo, and Zn IMNs after 6 and 12 weeks of implantation in rat femur (red: Zn; pink: Mo; yellow: O; green: Ca; violet: Ca; orange: C; cyan: N). (A2 and A3) Elemental composition of the surface corrosion products of Mo-Ti-Zr, Mo, and Zn IMNs after 6 and 12 weeks of implantation in rat femur determined by EDS. (B1) Representative optical images and SEM morphology of Mo-Ti-Zr, Mo, and Zn IMNs after removing surface corrosion products post-surgery at 6 and 12 weeks in rat femur. (B2) Corrosion rate of Mo-Ti-Zr and Mo IMNs compared to naked Zn IMNs after 6 and 12 weeks of implantation in rat femur. (C1) Representative images of three-point bending tests at 0, 6, and 12 weeks. (C2) Maximum bending force (left Y-axis) and corresponding bending force retention (right Y-axis) over time. (C3) Maximum bending stress calculated using the standard three-point bending equation. (For interpretation of the references to color in this figure legend, the reader is referred to the Web version of this article.)

The *in vivo* corrosion behavior of Mo-Ti-Zr-based IMNs implanted in a rat femoral fracture model after 6 and 12 weeks is illustrated in Fig. 11 as compared with the Mo and Zn ones. The pure Zn IMN bent at 6 weeks and fractured by 12 weeks, whereas both the Mo-Ti-Zr and Mo nails remained straight, exhibiting no bending or fracture (Fig. 11A1). Furthermore, the surfaces of Mo-Ti-Zr and Mo nails appeared dark black color at 6 weeks, with some metallic luster emerging by 12 weeks, likely due to readily detachment of products during the sample removal. The SEM images showed severe localized corrosion and accumulation of corrosion products on the bent Zn surface at 6 weeks, with products mainly composed of Zn, O, P, Ca, C, and N (Fig. 11A2). In contrast, Mo and Mo-Ti-Zr experienced milder corrosion; Mo had relatively uniform, sheet-like corrosion products with inter-sheet grooves, while Mo-Ti-Zr exhibited a denser, more uniformly distributed corrosion layer. Notably, Mo and Mo-Ti-Zr surfaces showed significantly higher Ca and P content than Zn, suggesting enhanced integration with bone tissue and increased calcium phosphate deposition. By 12 weeks, the corrosion products of Zn were loosely porous, while Mo displayed deeper grooves and partial detachment of corrosion products. The Mo-Ti-Zr surface remained relatively uniform, displaying sheet-like structures with minimal delamination, and had much higher Ca and P content than Zn and Mo content (Fig. 11A3). Nails after corrosion product removal (Fig. 11B1) showed severe localized corrosion on Zn, while Mo and Mo-Ti-Zr surfaces appeared smoother. Deep corrosion pits were visible on Zn, while Mo showed only minimal cracks, and Mo-Ti-Zr exhibited shallow corrosion marks, indicating a more uniform corrosion pattern. Moreover, *in vivo* corrosion rates further confirmed that the corrosion of Mo and Mo-Ti-Zr is less than that of Zn (Fig. 11B2). Specifically, Mo exhibited corrosion rates of ca. 18.33 μm/year and ca. 9.78 μm/year at 6 and 12 weeks, respectively; and the Mo-Ti-Zr IMN displayed corrosion rates of ca. 11.47 μm/year and ca. 10.98 μm/year at 6 and 12 weeks, respectively, obviously smaller than the former.

The mechanical integrity and strength of both Mo and Mo-Ti-Zr IMNs gradually declined with the prolonged implantation duration (Fig. 11C1), albeit the latter outperformed the former in its less declining rate. Specifically, the maximum bending force and stress of pure Mo showed a notably reduced value over 12 weeks, whilst the Mo-Ti-Zr IMNs exhibited significantly better mechanical retention with less reduced value, i.e. the bending force of Mo dropped to approximately 76.89 ± 3.05 % retention, whereas the Mo-Ti-Zr maintained an obvious higher value of 82.33 ± 2.45 % (Fig. 11C2). The bending stress followed a similar trend, with Mo-Ti-Zr retaining consistently higher values than Mo throughout the degradation period (Fig. 11C3). These results indicate that the Mo-Ti-Zr alloy possesses superior long-term mechanical retention under *in vivo* conditions over the pure Mo. It should be noted that the three-point bending test is effective to validate the mechanical performance of the IMNs samples, although their geometry and slightly smaller dimensions might impose some deviation from the supposed values if conducted under standard testing condition. The testing protocol is of more realistic meaning and valid enough for scientific comparison between the groups.

## 3 Discussion

Recent studies have identified metallic Mo as a promising BM, with significant potential in cardiovascular stent applications [16,19,27]. Moreover, substantial progress has been made in the development of Mo-based biodegradable electronic implants [[48], [49], [50], [51]]. Recent findings also suggested that metallic Mo could be a highly suitable material for bone implants [17,[20], [21], [22], [23], [24], [25]], although relevant reports remain limited in particular for Mo alloys. This study confirmed the excellent mechanical properties, uniform degradation mode in the bone microenvironment, and promising biocompatibility TZM (Mo-Ti-Zr) alloy, making it potential candidates for bone fracture fixation implants. Mechanical compatibility, biodegradability, and biocompatibility are essential requirements for application of BMs in bone repair [12]. Currently, three Mg-based bone implants are proved in the clinic use [9], and Zn-based craniofacial plates and interface screws are also being introduced into clinical practice [52], although both face still challenges and uncertain outcomes in a long-term course. Table 1 sums up and compares the main parameters of the Mo alloys with those of Mg and Zn based alloys on these key requirements.

**Table 1:** Comparison of key properties, including mechanical compatibility, biodegradability, and biocompatibility, between TZM Mo alloy based, Mg-based, and Zn-based biodegradable metals.

Key indicator		Mo and its alloys	Mg and its alloys	Zn and its alloys
Mechanical compatibility	Ultimate tensile strength (UTS; MPa)	>800 [15,55]	143–338 [56]	50–560 [12,57]
	Yield strength (YS)	>600 [55]	83–284 [56]	126-389 [12]
	Elongation to failure (%)	0–30 [55]	3–25 [56]	0–75 [57]
	Compressive yield strength (CYS; MPa)	>400 [58]	21-170 [59]	82–454 [60]
	Hardness (HV)	200–300 [58]	40–50 [12]	32–216 [60]
	Elastic modulus (GPa)	200–250 [55]	41–45 [61]	94–110 [62]
Biodegradability	*In vitro* degradation rate (Electrochemical measurements and long-term immersion; μm/year)	0.60–33.6 [21]	122-13184 [56]	100–300 [21,57,66]
	*In vivo* degradation rate (Bone implant microenvironment; μm/year)	9.78–18.33 ^[This study]^	>100 [56]	65–220 [67]
	*In vivo* degradation rate (vascular tissue microenvironment; μm/year)	2–55 [20]	>100 [56]	3–50 [20,68]
	Degradation mode	Uniform ^[This study]^	Localized + Pitting [57]	Uniform + localized [57]
	Major degradation products (Gaseous)	No	H_2_	No
	Major degradation products (Soluble)	MoO_4_^2−^; HMoO_4_^−^ [21]	Mg^2+^; OH^−^	Zn^2+^; OH^−^
	Major degradation products (Solid and solubility in neutral environment)	MoO_2_ (insoluble); MoO_3_ (sparingly soluble); Calcium phosphates (K_sp_ = 2.07 × 10^−33^)	Mg(OH)_2_ (K_sp_ = 5.61 × 10^−12^); MgO (K_sp_ = 1.8 × 10^−11^); Mg_3_(PO_4_)_2_ (K_sp_ = 1 × 10^−24^); Calcium phosphates (K_sp_ = 2.07 × 10^−33^)	Zn(OH)_2_ (K_sp_ = 4.5 × 10^−17^); ZnO (K_sp_ = 3.0 × 10^−15^); Zn_3_(PO_4_)_2_∙4H_2_O (K_sp_ = 9.1 × 10^−33^); Calcium phosphates (K_sp_ = 2.07 × 10^−33^)
Biocompatibility	Essential elements on bone metabolism	No	Yes	Yes
	Serum concentration (mmol/L)	6.25 × 10^−5^–5.63 × 10^−3^ [71]	0.73–1.06 [57]	0.012–0.017 [57]
	Dietary average daily intake (mg)	0.109 [71]	300–400 [57]	8–11 [57]
	Recommended daily intake (mg/d)	0.083–0.247 [72]	375–500 [57]	2-15 [57]
	Cytotoxicity threshold (HUVECs)	0.5 mM (47.98 μg/mL) ^[This study]^	10 mM (243.05 μg/mL) [73]	0.08 mM (5.2 μg/mL) [75]
	Cytotoxicity threshold (BMSCs)	0.5 mM (47.98 μg/mL) ^[This study]^	10 mM (243.05 μg/mL) [74]	0.10 mM (6.8 μg/mL) [76]
	LD50 (mg/kg)	180–350	>5000	200–400
	Angiogenesis	Yes	Yes	Yes
	Osteogenesis	Yes	Yes	Yes
	Anti-osteoclastogenesis	Yes	Yes	Yes

### 3.1 Mechanical properties

For BM materials, the primary consideration is the duration of device functionality, which depends on their mechanical compatibility with host tissues. The implant should provide temporary mechanical support during tissue regeneration and subsequently degrade at an appropriate rate (200–500 μm/year) for the human body until complete dissolution [53]. For IMNs used in fracture fixation, at least 3–6 months of fully mechanical support are required [54]. The mechanical properties of BMs are directly related to their degradation kinetics, and current BMs still face challenges in balancing mechanical compatibility and degradation rates. Mg-based IMNs, for instance, would lose their strength and fracture within approximately 1 month due to rapid degradation, making them unsuitable for use in load-bearing sites [11]. Pure Zn-based IMNs bent at 4 weeks and fractured completely by 16 weeks post-implantation in femur of rats [33,34]; although some Zn alloys have exhibited higher strength, correlation between their degradation and mechanical stability is yet to be confirmed *in vivo* [12]. In contrast, Mo-based metals, including Mo and Mo-Ti-Zr, have demonstrated significant advantages in this regard (Table 1). Mo-based alloys possess much higher UTS and yield strength than Mg and Zn as well as superior mechanical ductility, hardness, and elastic modulus, which can ensure sufficient mechanical integrity during the healing process [12,15,[55], [56], [57], [58], [59], [60], [61], [62]]. Previous studies indicate that pure Mo retained over 350 MPa in UTS even after 6 months of immersion in simulated body fluid, meeting the mechanical requirements of most orthopedic implants, including IMNs [17]. In our *in vivo* implantation study, the Mo and Mo-Ti-Zr IMNs maintained favorable mechanical retention, with the maximum bending strength remaining at approximately 76 % and 83 %, respectively, after 12 weeks of implantation. The Mo-Ti-Zr alloy selected in this study outperformed even the pure Mo in terms of mechanical properties, providing ample strength (Table S5, Supplementary Materials). It is worth to note that the elastic modulus of Mo and Mo-Ti-Zr alloys was much higher than that of cortical bone, typically ranging from 4 to 30 GPa [55], which might cause the notorious stress-shielding effect. Nonetheless, given that the internal fixation implants might be not as sensitive as the permanent bone prostheses like hip joint on this regard and their mechanical integrity may decline over time, this might be of less concern as expected.

### 3.2 Mild degradation of Mo-Ti-Zr

The corrosion mechanism of Mo in physiological environments has been extensively investigated in previous studies [[15], [16], [17], [18],^20^,^21^,^30^]. However, to the best of our knowledge, the corrosion behavior of Mo-Ti-Zr alloys as BMs has not yet been investigated. Generally, the alloying leads to a refined grain structure, reduced grain boundary defects, and slower corrosion, preventing localized and pitting corrosion [63]. The corrosion behavior of Mo is closely associated with the types and physicochemical properties of its surface oxides [15]. During degradation, the surface oxide layer of metallic Mo transitions from a low oxidation state (e.g., MoO_3-x_, 2 < x < 3) to an intermediate state (such as MoO_2_) and eventually to a high oxidation state (Mo_2_O_5_ and MoO_3_) in a dynamic process involving dissolution [64] (Fig. 3E1 and Fig. 3E2). At the initial immersion stage, oxygen reduction occurs at the cathode, while Mo oxidation occurs at the anode, as described by the following equations:

(Eq. 1) Anodic: Mo + 2H_2_O → MoO_2_ + 4H^+^ + 4e^−^

(Eq. 2) Cathodic: O_2_ + 4H^+^ + 4e^−^ → 2H_2_O

Over time, the oxidation state of the Mo surface oxides gradually increases, and dissolves to form molybdate ions (MoO_4_^2−^) or hydrogen molybdate ions (HMO_4_^-^), as shown in the following equations:

(Eq. 3) Mo + 4H_2_O → MoO_4_^2−^ + 8H^+^ + 6e^−^

(Eq. 4) 2MoO_2_ + H_2_O → Mo_2_O_5_ + 2H^+^ + 2e^−^

(Eq. 5) 2MoO_2_ + 2H_2_O → 2MoO_3_ + 4H^+^ + 4e^−^

(Eq. 6) Mo_2_O_5_ + H_2_O → 2MoO_3_ + 2H^+^ + 2e^−^

(Eq. 7) MoO_2_ + 2H_2_O → MoO_4_^2−^ + 4H^+^ + 2e^−^

(Eq. 8) MoO_3_ + H_2_O → HMoO_4_^−^ + H^+^

(Eq. 9) MoO_3_ + H_2_O → MoO_4_^2−^ + 2H^+^

Mo oxides exhibit semiconductor behavior; as the oxidation state increases, the bandgap widens, resulting in distinct colors. MoO_2_, with a semi-metallic nature and bandgap of 0.2–0.3 eV, typically appears black. Mo_2_O_5_ has a bandgap of 1.0–2.7 eV, presenting a blue or blue-green color, while MoO_3_, a wide-band-gap semiconductor with a band gap of 2.7–3.2 eV, appears white or pale yellow [64]. The observed color changes on Mo and Mo-Ti-Zr surfaces, along with XPS and XRD results, confirmed this transformation of oxides, as reported in previous studies [15,21].

From the perspective of corrosion modes, previous studies have observed minor pitting on pure Mo discs after immersion for 28 days in inflammatory solutions (containing H_2_O_2_), but this effect is negligible compared to the degradation observed in Mg, Zn, and Fe [21]. The corrosion product layer on Mo surfaces is normally uniform and dense. For example, commercial Mo foil immersed in c-simulated body fluid-Ca solution for 28 days developed a corrosion layer of approximately 1–1.5 μm thick [65]. Similarly, polished Mo wire immersed under the same conditions formed a 2.8-μm thick corrosion layer [18], while Mo discs immersed in phosphate-buffered saline (PBS) containing H_2_O_2_ and bovine serum albumin for 28 days form a corrosion layer with a thickness of 3.7–5.2 μm [21]. Herein, after 56 days of immersion, the thickness of the corrosion layer for Mo-Ti-Zr and Mo was ca. 1.21 μm and ca. 2.52 μm, respectively, with Mo-Ti-Zr displaying greater corrosion uniformity. Note that the corrosion products on Mo-Ti-Zr surfaces exhibited a discontinuous lamellar crystal structure, with fissures or cracks between the lamellae increasing over time, as observed in multiple studies [[15], [16], [17], [18],^20^,^21^,^30^]. This may partly result from the Mo-Ti-Zr sintering process, which produces irregular grain structures that lead to intergranular stress, causing corrosion occur preferentially along grain boundaries during corrosion product formation. Additionally, Mo oxides have anisotropic properties, favoring deposition along specific crystallographic planes (e.g., cleavage or weak bonding planes), and the high rigidity of the lamellar crystals contributes to discontinuities between these layers [64]. More importantly, the Mo-Ti-Zr surface showed virtually no pits, further confirming its more uniform corrosion pattern.

Degradation rate is another critical indicator for BM implants. Pure Mo in forms such as foils, plates, rods, and wires has been shown to have a corrosion rate of approximately 0.6–33.6 μm/year in neutral physiological environments (Table 1), which is lower than that of Mg, Zn, and Fe [20,21,56,57,[66], [67], [68]]. According to the literature, the degradation rate of Mo *in vivo* ranges from 2 to 55 μm/year [15,20], and the corrosion rates of Mo and TZM IMNs used herein were within this range, although these rates remained about ten times lower than the seemingly ideal degradation rate for bone implants (200–500 μm/year) which was based on the commonly BMs that have much poor mechanical properties and therefore thicker design. Further, both *in vitro* and *in vivo* degradation rates were measured herein, revealing substantial differences. After 56 days of immersion, weight-loss calculations indicated an *in vitro* degradation rate of 40–50 μm/year, while polarization curve estimates suggested a rate of 2000–3500 μm/year. *In vivo*, the degradation rate of IMNs implanted for 12 weeks was ∼10 μm/year. The differences between the *in vitro* and *in vivo* rates were attributed to the direct exposure of *in vitro* samples to the corrosion medium, where there is unrestricted exchange of electrolytes and oxygen — an essential factor in the corrosion of Mo and its alloys. Additionally, periodic renewal of the corrosion medium maintains a high level of corrosive ions (e.g., Cl^−^). In contrast, *in vivo*, IMNs are largely in contact with surrounding bone tissue, with limited contact with the bone marrow medium, thereby being restricted regarding oxygen and electrolyte exchange. Consequently, the *in vivo* degradation rate is more representative of the clinical conditions, making it preferable for assessing the degradation timelines of BMs. It is noteworthy that the complete degradation of Mo and TZM IMNs in the human body would not be achievable within a reasonable time frame based on the *in vivo* corrosion rate observed in this study from the standpoint of conventional BMs. Likewise, current evidence suggests that the maximum *in vivo* (including in cardiovascular and bone microenvironments) degradation rate for Mo and its alloys does not exceed 55 μm/year [20,21]. Encouragingly, the superior strength and stiffness of Mo, particularly in Mo-Ti-Zr alloys, support designs that utilize thinner, smaller structures while maintaining the required mechanical strength, offering probably new criteria for the degradation rate in practical applications.

As BM implants, the solubility of the corrosion products is also a key factor that requires close attention. For Mg and Zn, the released metal ions and hydroxide ions are readily absorbed or metabolized by tissues, while their insoluble corrosion products are dissolved and absorbed within an acceptable time frame in bone environments [12]. In contrast, Mo oxides might have lower solubility than the hydroxides and phosphates of Zn and Mg. The solubility of MoO_3_ and MoO_2_ depends on the formation of MoO_4_^2−^ or HMoO_4_^−^ ions. Nevertheless, it was reported the soluble Mo ions and MoO_4_^2−^ released from Mo-Ti-Zr implants can be absorbed, metabolized, and utilized by surrounding cells or tissues [20,69]. More importantly, small insoluble degradation particles such as MoO_2_ and MoO_3_ may be phagocytosed and transported by macrophages [20,69]. Moreover, the corrosion products on Mo surfaces promoted calcium phosphate deposition. *In vivo* corrosion results showed that Ca and P content on the surface of Mo-Ti-Zr IMNs was significantly higher than that on pure Zn IMNs. This effect may be attributed to the negatively charged Mo oxide layer, which preferentially attracts Ca^2+^ [70]. Such calcium phosphate deposition may facilitate subsequent osteogenesis. In addition, Zr released from Mo–Ti–Zr alloys in physiological environments exists both in ionic form (mainly as hydrated or hydroxyl-coordinated charged species) and in other mixed forms (such as Zr(OH)_4_, hydrated zirconia colloids/nano-oxides, and complexes with proteins), which, although having relatively low overall solubility, can still interact with extracellular proteins and cells and may contribute to the observed osteogenic and angiogenic responses.

### 3.3 Favorable angiogenic and osteogenic ability of Mo-Ti-Zr

Undoubtedly, biocompatibility is a prerequisite for evaluating potential biomaterials. Mo-Ti-Zr alloys exhibited excellent biocompatibility in both *in vivo* and *in vitro* settings. The results of this study show that Mo-Ti-Zr is highly compatible with HUVECs and BMSCs, causing no cellular damage or apoptosis. Mo-Ti-Zr was also well-tolerated by tissue at fracture sites, producing no hyperplasia, necrosis, or excessive inflammatory response. Furthermore, the samples did not induce hemolysis or alter blood parameters *in vitro* and *in vivo*.

Mo is an essential trace element for nearly all organisms, serving as a catalytic center in several enzymes, and plays a crucial role in promoting cellular behavior and function [20,26]. The recommended daily intake of Mo is 0.083–0.247 mg, significantly lower than that of Zn (8–11 mg) or Mg (200–400 mg), with considerable differences in serum levels and dietary average daily intake thresholds compared to Zn and Mg (Table 1) [57,71,72]. In both direct culture on Mo-Ti-Zr surface and incubation with 100 % extraction media, no cytotoxicity was observed toward HUVECs or BMSCs and even promoted cell proliferation. Further, the effects of Mo ion (Mo^5+^) on HUVECs and BMSCs were dependent on concentration and culture time, consistent with previous findings [23,29,65,73,74]. However, when Mo ion concentrations exceed 0.5 mM (ca. 48 μg/mL), adverse cellular effects may occur; this threshold is significantly higher than the tolerance level for Zn^2+^ (5.2 μg/mL for HUVECs; 19.65 μg/mL for BMSCs) [75,76]. The Mo ion concentration in Mo-Ti-Zr extraction media remained well below this threshold and, considering that degradation in the bone implantation environment occurs in a static state, the actual Mo ion release is likely lower *in vivo* than in *in vitro* measurements, although this cannot be measured in real time. Moreover, the soluble Mo ions and MoO_4_^2−^ released from Mo-Ti-Zr implants can be absorbed, metabolized, and utilized by surrounding cells or tissues [20]. Solid degradation products, such as MoO_2_ and MoO_3_, also have good cellular compatibility both *in vivo* and *in vitro* [20,23]. *In vivo* results confirmed the formation of abundant new bone around Mo-based IMNs, with no evidence of thick fibrous layers around the implants, which is often observed in the early stages of implantation of Zn-based and Mg-based materials due to the accumulation of high volumes of corrosion products [10,12]. Previous studies also indicated that Mo can promote new bone formation at bone defect sites [25].

Organ histology results revealed that Mo-Ti-Zr corrosion products and ions did not accumulate in major organs, with no pathological changes observed, particularly in the kidneys, which are the primary pathway for Mo excretion [16]. According to previous studies, excessive Mo intake (80 mg/kg/day) may cause toxicity, leading to renal dysfunction [77]. However, this concentration is several orders of magnitude higher than the release of Mo ions from Mo-based bone implants.

The repair of bone fractures relies critically on vascularization with participation of bio-active metallic ions. Our *in vitro* assays on extracts samples depicted that both the Mo and particularly the Mo-Ti-Zr alloy favored pro-angiogenic activity (Fig. 4F3), in tandem with the significantly up-regulated angiogenesis-related gene expression, compared to the control and AZ31 groups. This implies the released metallic ions play a crucial part in governing this bio-response. More important, as the ICP analysis revealed the Mo-Ti-Zr and Mo extracts differed in the level of Mo and molybdate ions release. It must be emphasized that it has been well-established such bio-active metallic ions associated reactions are always concentration-dependent, namely an optimal concentration range exits and too high concentration usually leads to adverse effects [69]. Based on the fact that the detected concentration of Mo ions in our TZM extract was apparently smaller than that of the pure Mo group, it can be safely concluded this difference in the concentration might account for their different performances, although both fell below the cyto-toxic threshold (<1 mM) as we validated. Such dose-dependent effect should apply to molybdate ions, which must be in line with its Mo ions release according to the corrosion electrochemistry. It is also worth noting Zr ions, as detected out in its extracts, may contribute partly to the enhanced angiogenesis of TZM as it has been reported to perform such functionality [78]. Nonetheless, its degree of this contribution might be not as high as the Mo or Molybdate ions due to its minimal amount compared to the latter. Whether there is a synergistic effect between them remains also unclear. Likewise, the surface characteristics including the physicochemical states, oxides composition and morphology may contribute partially to the better angiogenesis found on the TZM than the Mo particularly in the *in vivo* circumstance, as it is evident the TZM presented some more uniform corrosion morphology with likely more multi-type oxides such as titanium and zirconium oxides [31]. Further, the above explanations are supported well by their molecular expression results. First, according to the immunofluorescence and WB results, the canonical Wnt-β-catenin signaling pathway was activated more on the TZM than on Mo, which is known to promote endothelial proliferation, migration, and survival via regulation of VEGF, Angpt1, and delta-like ligand 4 (DLL4) [44]. In particular, VEGF plays a central role by up-regulating the downstream genes such as endothelial nitric oxide synthase, focal adhesion kinase, and matrix metalloproteinases [43]. Note Mo-containing compounds such as molybdates take part also in these bio-reactions, e.g. MoO_4_^2−^ ions has been reported to trigger the HIF-1α pathway, contributing to angiogenic effect as well [79]. Nonetheless, it appears that these expressions behave also as a function of concentration for there seemed to be some conflicting results in the literature regarding their role in angiogenesis. Especially, for their compounds in solid state like MoO_3_ nanoparticles (300 μg) and Na_2_MoO_4_·2H_2_O (0.5 M, 200 μL) with very high concentration they performed anti-angiogenesis in the chorioallantois membrane assays [80,81]. Another study incorporating MoO_3_ into mesoporous bioactive glass showed also retarded endothelial cell migration at concentrations exceeding 60 μg/mL [82]. However, all their concentrations are much higher (orders of magnitude) than those in our study, so these results cannot be directly translated into our findings. Considering the importance of concentration when addressing these bio-activities, further investigation is needed to clarify their distinctive contributions thereof with more advanced techniques such as pathway inhibitors or gene silencing method in spite of that our results looked promising.

The osteogenic performance of the Mo-Ti-Zr and Mo was superior to that of the control group, with the Mo-Ti-Zr exhibiting particularly enhanced effects on bone healing, which can be still mainly attributable to the released degradation products. The proposed mechanisms underlying this osteogenic enhancement are illustrated in Scheme 1 and Fig. 7F. According to transcription sequencing, immunofluorescence staining, and WB analyses, Mo extracts promoted osteogenesis by activating the MAPK–ERK and cAMP–PKA signaling pathways, which up-regulated the key osteogenesis-related proteins, such as Runx-2, OSX, OCN, ALP, and OPN. Notably, compared to pure Mo, the Mo-Ti-Zr extracts appeared to activate the PI3K–Akt signaling pathway and be associated with higher expression levels of OSX, OCN, and BMP-2. The three signaling pathways work in a parallel and mutually responsive way. Briefly, the MAPK–ERK and cAMP–PKA signaling pathways play crucial roles in bone repair [41,45]. MAPK–ERK is essential in regulating cell proliferation, differentiation, and apoptosis and is integral to bone metabolism, up-regulating osteogenesis-associated genes, including *ALP, COLI*, *Runx-2*, and *OPN* [41]. The cAMP–PKA pathway is involved in multiple physiological processes, including cell proliferation, differentiation, and apoptosis [45]; PKA activation promotes the up-regulation of osteogenic factors such as Runx-2 and OCN, thus facilitating osteoblast proliferation and differentiation [45]. Furthermore, crosstalk between cAMP and MAPK might enhance osteoblasts proliferation [83]. The PI3K–Akt signaling pathway plays a crucial role in regulating cellular functions, such as survival, proliferation, and migration, and is involved in mitosis [40]. It promotes bone regeneration and remodeling by up-regulating osteogenesis-related proteins, including osteoprotegerin (OPG) and BMP-2 [40]. It has been found that Mo ions or MoO_3_ up-regulate ALP and BMP-2 expression and accelerate BMSCs mineralization [79,82], as the same as we observed in this study. Again, these pathways and their degree of activation might be still concentration-dependent. Consistently, our concentration–gradient experiments revealed a biphasic response of BMSCs to Mo ions, in which low to moderate Mo levels led to better cell viability, ALP activity, and mineralized nodule formation, whereas too high concentration caused cytotoxicity. Previous report indicated that Mo ion concentrations up to 31.65 ± 0.57 mg/L favored osteogenesis, with a range from 2 to 6 mg/L having the most pronounced effect [29]. It can be seen interestingly that the Mo concentration in the αMEM extracts in this study fell right into this range. It seems the lower side of this concentration scope favored more the related expressions, which explains probably the reason why TZM outperformed pure Mo on the osteogenesis as well. In fact, there are more evidence on the improved bone healing caused by Mo related substances. For examples, the Mo ions activate the PI3K–mTOR pathway in macrophages, promoting M2 polarization, which contributes to hair follicle regeneration [84]. MoO_4_^2−^ ions also enhance mitochondrial function in macrophages, induce M2 polarization, and support periodontal wound healing [28]. This is the same with Zr ions. The graded Zr ion treatments showed that low-to-moderate Zr concentrations are associated with improved BMSC viability and increased the expression of osteogenic markers, whereas higher concentrations than micromolar level rendered markedly poorer viability and mineralization. In short, the Mo-Ti-Zr outperformed pure Mo on the osteogenic performance may account mainly to their differences in the released Mo ion and Molybdates ions in terms of concentration and partially Zr ions as possible. It must be emphasized that the trace elements of Ti and Zr are generally considered biologically inert, but this is more the case for the bulk form of two materials as metal. For Zr ions released, they still exert osteogenic effects as some other bio-active metallic ions, potentially involving the activation of pathways such as BMP–Smad or PI3K–Akt, which regulate osteoblast proliferation [85,86]. Further, there may be still potentially some synergistic interactions between Mo and Zr ions when they serve to regulate the osteogenesis, as proposed in previous studies on Mo-based materials [34,79], although this needs to be validated further and their underlying mechanisms elucidated.

### 3.4 Limitations and perspectives

This study has thoroughly explored the application of TZM IMNs in fracture fixation as compared with pure Mo, yielding promising results, but the potential for stress shielding due to the relatively high elastic modulus of the TZM requires further investigation. Additionally, understanding the relationship between changes in mechanical properties (such as elastic modulus) and the progression of corrosion and degradation of Mo-based IMNs during implantation remains an urgent research focus.

The degradation rate of the TZM alloy as well as Mo was considerably lower than anticipated (almost an order of magnitude lower). For bone implants, an ideal degradation rate should align with the rate of bone healing. Therefore, further optimization of IMN design is needed to align with the degradation profile. Strategies could include developing hollow or porous structures to reduce material use while maintaining strength and better matching the expected degradation timeline. For potential biological mechanisms of the angiogenic and osteogenic properties of the TZM alloy and its advantageous performance over pure Mo, and we identified its pathways such as Wnt/β-catenin and PI3K-Akt. Further studies using more pathway-specific inhibitors or siRNA knockdown would be of necessity to clarify more the causal cell signaling pathways in the relevant cascades of bio-responses. Moreover, the influence of the TZM alloy on other biological effects, such as modulation of the bone immune microenvironment and regulation of osteoclast phenotypes, also requires investigation. In addition, our results in particular of the *in vitro* assays cannot be translated directly into the clinical practice, future work will expand the spectrum of investigations. For example, although our 50 % extracts selected for the bio-evaluations served to distinguish their difference in shaping the cells behavior with no interruption of toxicity, it deviates still from some more realistic dilutions such as 6–10 % as recommended in some studies to better-mimic the physiological ion exposure levels -[87]. In future work, this should be considered as well to better-align with clinical relevance. Importantly, studies should assess the performance of TZM implants in large animal models, such as beagles and goats, to comprehensively evaluate their clinical potential. It is worth noting that further detailed characterization of the TZM bio-metal is necessary in the future studies. For example, surface 3D profilometry can be applied to analyze the localized corrosion behavior in more details. An alternative would be 3D imaging techniques such as micro-CT for following-up implanted bio-metals *in vivo*, but it must be emphasized that does not work well to TZM alloys because of molybdenum's high atomic number causing apparent X-ray artifacts according to our experience. Mo might be MRI compatible, whereas it needs to be validated on TZM in future studies.

## 4 Conclusions

This study comprehensively evaluated the mechanical compatibility, corrosion/degradation behavior, and biocompatibility of commercially-available TZM (Mo-Ti-Zr) as a superior biodegradable metal over other or pure Mo candidates for intramedullary nail (IMN) through both *in vitro* and *in vivo* assays, and elucidated the underlying molecular mechanisms in particular for their promotion of angiogenesis and osteogenesis for bone regenerative repairing. The TZM alloy exhibited uniform degradation patterns and minimal Mo ion release compared with pure Mo. The TZM IMNs potentially activated the Wnt–β-catenin signaling pathway, which may contribute to up-regulating angiogenesis-related gene expression and therefore enhancing vascularization. Additionally, transcriptomic analysis indicated possible involvement of MAPK–ERK, cAMP–PKA, and PI3K–Akt pathways in promoting osteogenesis, which were partially validated by WB. Remarkably, the TZM IMNs were verified to be capable of facilitating new bone formation to accelerate fracture healing in rat femur fracture models while they degraded gradually. In short, the commercially-available TZM Mo alloy behaves superb as biodegradable metal for bone-fracture healing IMN, connotating promisingly in more load-bearing temporary biomedical implants applications, and further studies are needed to provide additional insights.

## CRediT authorship contribution statement

**Junyu Qian:** Writing – original draft, Methodology, Investigation, Funding acquisition, Formal analysis, Data curation, Conceptualization. **Yukun Zhou:** Methodology, Investigation, Formal analysis, Data curation. **Zhenhai Xie:** Methodology, Formal analysis, Data curation. **Jinjing Liu:** Methodology, Formal analysis, Data curation. **Ping Li:** Resources, Investigation, Data curation. **Wenjie Tao:** Methodology, Data curation. **Yuanhao Wang:** Methodology, Data curation. **Fei Gao:** Resources, Methodology, Formal analysis. **Hui Zeng:** Supervision, Resources. **Deli Wang:** Supervision, Resources. **Haotian Qin:** Writing – review & editing, Methodology, Conceptualization. **Yingqi Chen:** Writing – review & editing, Supervision, Conceptualization. **Guojiang Wan:** Writing – review & editing, Supervision, Project administration, Funding acquisition, Conceptualization.

## Declaration of competing interest

The authors declare that they have no known competing financial interests or personal relationships that could have appeared to influence the work reported in this paper.

## Data Availability

Data will be made available on request.
