# Supplementary material for: TZM Mo alloy behaves superb as biodegradable metal for bone-fracture healing intramedullary nail implant
Source: Mater Today Bio. 2026 Jan 12;37:102794. doi: 10.1016/j.mtbio.2026.102794 (PMC12858364; doi:10.1016/j.mtbio.2026.102794)
Supplement: Multimedia component 1 [file mmc1.docx]

**TZM Mo Alloy Behaves Superb as Biodegradable Metal for Bone-fracture Healing Intramedullary Nail Implant**

Junyu Qian ^a, b, c1^, Yukun Zhou ^a1^, Zhenhai Xie ^a, b^, Jinjing Liu ^a^, Ping Li ^d^, Wenjie Tao ^a^, Yuanhao Wang ^a, b^, Fei Gao ^a^, Hui Zeng ^e^, Deli Wang ^b^, Haotian Qin ^b*^, Yingqi Chen ^b, f*^, Guojiang Wan ^a*^

^a^ Institute of Biomedical Engineering, College of Medicine, Key Laboratory of Advanced Technologies of Materials, Ministry of Education, Southwest Jiaotong University, Chengdu 610031, China

^b^ National & Local Joint Engineering Research Centre of Orthopaedic Biomaterials, Department of Bone & Joint Surgery, Peking University Shenzhen Hospital, Shenzhen, 518036, China

^c^ Shenzhen Peking University-The Hong Kong University of Science and Technology Medical Center, Peking University Shenzhen Hospital, Shenzhen, 518036, China

^d^ School and Hospital of Stomatology, Guangdong Engineering Research Center of Oral Restoration and Reconstruction, Guangzhou Medical University, Guangzhou, China

^e^ Department of Orthopedics, Medical Innovation Technology Transformation Center of Shenzhen Second People’s Hospital, Shenzhen Second People’s Hospital, Shenzhen, Guangdong, China

^f^ Medical Innovation Technology Transformation Center of Shenzhen Second People’s Hospital, Shenzhen Second People’s Hospital, Shenzhen, Guangdong, China

^1^ These authors contributed equally to this work.

^*^**Corresponding author 1:** Professor & PhD. Guojiang Wan

Institute of Biomedical Engineering, College of Medicine, Key Laboratory of Advanced Technologies of Materials, Ministry of Education, Southwest Jiaotong University, Chengdu 610031, P.R. China

**E-mail**: guojiang.wan@home.swjtu.edu.cn

^*^**Corresponding author 2:** Professor & PhD. Yingqi Chen

Department of Bone & Joint Surgery, National & Local Joint Engineering Research Center of Orthopaedic Biomaterials, Peking University Shenzhen Hospital, Shenzhen 518036, P.R. China

E-mail: yingqi.chen@szu.edu.cn

^*^**Corresponding author 3:** PhD. Haotian Qin

Department of Bone & Joint Surgery, National & Local Joint Engineering Research Center of Orthopaedic Biomaterials, Peking University Shenzhen Hospital, Shenzhen 518036, P.R. China

**E-mail:** qht@pku.edu.cn

1. **Experimental Section**

**1.1 Preparation of pure Mo and Mo-Ti-Zr alloy specimens**

High-purity Mo (99.99%) and commercially TZM Mo alloy (Titanium-Zirconium-Molybdenum, Mo-Ti-Zr; 99.99%) were purchased from Qinghe Xiuhang Metal Materials Co., Ltd., China, and cut into disc specimens with a diameter 10 mm and a thickness of 1.8 mm. Following the commercial TZM specification, the alloy composition was fixed at Ti 0.4–0.6 wt.%, Zr 0.08–0.12 wt.%, and C 0.02–0.03 wt.% to achieve grain refinement and stronger grain boundaries in Mo and to ensure biomedical acceptance; this microstructural design underpins the more uniform corrosion and mechanical retention observed for TZM in this study. The disc samples were mechanically ground with sandpaper in the following sequence: 180#, 300#, 1200#, and 2000# grit. Thereafter, the samples were successively subjected to ultrasonic washing with deionized water, ethanol (purity ≥ 99.7%, Chengdu Kelong Chemical Co., Ltd., Chengdu, China), and acetone (purity ≥ 99.5%, Chengdu Kelong Chemical Co., Ltd., Chengdu, China). The samples were washed three times in each solution, with each washing lasting 5 minutes. The cleaned samples were dried in a vacuum oven (Tianjin Taisite Instrument Co., Ltd., China) for further investigation.

**1.2 Material characterization**

The surface morphology and elemental distribution of Mo and Mo-Ti-Zr were imaged and determined by a tungsten filament scanning electron microscope (SEM; JSM-IT500, JEOL, Japan) and a coupled energy dispersive spectroscope (EDS; OXFORD X-Max 80, UK) at 30 A beam current with 15 kV acceleration voltage. The microstructure of Mo and Mo-Ti-Zr was observed utilizing a metallurgical microscope (AxioCam ERc5s, ZEISS, Oberkochen, Germany) and a SEM. Briefly, the ground samples were further polished using a metallographic polisher (LMP-3S, Huaxia Measuring Instrument Technology Co., Ltd., Beijing, China) with a polishing cloth and 1.5 μm polishing paste. Subsequently, a mixed solution in a 2:1 ratio of 15% potassium ferricyanide (Analytical Grade, Chengdu Kelong Chemical Co., Ltd., Chengdu, China) and 15% sodium hydroxide (purity ≥ 98%, Chengdu Kelong Chemical Co., Ltd., Chengdu, China) was applied to the surface of polished Mo and Mo-Ti-Zr for 90 seconds ^[1]^. The samples were imaged after cleaning and drying. The surface phase composition and crystal structure of Mo and Mo-Ti-Zr were determined by X-ray diffraction (XRD; X’pert PRO, PANalytical, Almelo, Netherlands) with Cu-Kα radiation at a scanning angle of 2° under a 2θ range of 10° to 80°.

- 1. ***In vitro* corrosion behavior evaluation**

*1.3.1 Transient electrochemical measurement*

The transient electrochemical measurements, including open circuit potential (OCP), potentiodynamic polarization (PDP) curves, and electrochemical impedance spectra (EIS), were performed at 37 °C utilizing an electrochemical workstation (IM6, Zahner, Germany) in Hank’s solution (8 g/L NaCl, 0.4 g/L KCl, 0.15 g/L Na_2_HPO_4_⋅12H_2_O, 0.14 g/L CaCl_2_, 0.1 g/L MgSO_4_⋅7H_2_O, 0.1 g/L, MgCl_2_⋅2H_2_O, 0.06 g/L KH_2_PO_4_, 1 g/L D-glucose, 5.6 wt% NaHCO_3_) at a pH of ca. 7.4 ^[2, 3]^. All chemical reagents were of analytical grade and were purchased from Chengdu Kelong Chemical Co., Ltd., Chengdu, China. A three-electrode cell system, including reference electrode (saturated calomel electrode), working electrode (research samples, exposed area ca. 0.79 cm^2^), and counter electrode (platinum, 1.5 × 1.5 cm), was selected to conduct the electrochemical test. Before the test, the backside of the test samples was connected with copper wire and sealed with silicone rubber. The OCP of Mo and Mo-Ti-Zr was monitored for 8 hours and values were recorded every three seconds. After 15 min of stabilization in Hank’s solution, the PDP curves of Mo and Mo-Ti-Zr were tested at a potential range of -1.1 to 0.3 V and a scanning rate of 1 mV/s. The self-corrosion potential (*E*_corr_) and self-corrosion current density (*i_c_*_orr_) were obtained from the PDP curves by a Tafel linear extrapolation method ^[4, 5]^. The corrosion rate (*P_i_*) was calculated according to the following equation ^[6]^:

$\text{P}_{\text{i}}\text{=3.27×}\text{10}^{\text{-3}}\frac{\text{i}_{\text{corr}}\text{EW}}{\text{ρ}}$ (Eq. 1)

Where *P_i_* is the corrosion rate; *i_c_*_orr_ represents the self-corrosion current density; *EW* stands for the weight equivalent (determined by the relative atomic mass (95.95 g/mol) and valence state (commonly used 6) of the element Mo; and *ρ* is the material density of Mo (10.28 g/cm^3^). Namely, *P_i_* = 5.09*i*_corr_.

For the EIS tests, the spectra were recorded under a sinusoidal perturbation voltage of 10 mV, covering a frequency range from 200 kHz to 0.01 Hz. The data were fitted using ZSimpWin software (version 3.60, EChem Software, MI, USA) to obtain the kinetic and thermodynamic parameters. A minimum of four parallel samples were tested for each group.

*1.3.2 Long-term immersion test*

The *in vitro* long-term immersion test was conducted in Hank’s solution over 7, 14, 28, 42, and 56 days at 37 ± 0.5 °C, at pH 7.4. Samples were sealed with epoxy resin to expose only the research surface and immersed at a solution volume-to-surface area ratio of 20 mL/cm², in accordance with ASTM-G31 ^[7]^. The samples were placed in sealed centrifuge tubes to avoid the effects of gases, such as CO_2_, on corrosion. The solution was refreshed every 3 days to simulate the fluid conditions in the body. Samples were retrieved at each designated time point (7, 14, 28, 42, and 56 days) for electrochemical measurements, as described in Section 1.3.1; note the potential range of PDP test is -1.5 to 0.3 V. Surface corrosion morphology at every time point was observed using the same optical microscope (SZX7-1093, Olympus, Tokyo, Japan) and SEM. After 56 days of immersion, the cross-sectional morphology was imaged by SEM. Elemental distribution, including Mo, O, P, and Ca, was assessed using SEM coupled with EDS. Phase composition of the corrosion products was determined by XRD, with Cu-Kα radiation at a scanning rate of 8 °/min, covering a 2θ range from 20° to 80°. Chemical compositions and bonding states in the surface corrosion products were determined by X-ray photoelectron spectroscopy (XPS; XSAM800, Kratos, Ltd, UK) under an Al-Kα X-ray source and a pressure of 2 × 10^−7^ Pa, with a current at 15 mA and voltage at 12 kV. The binding energy of C 1*s* at 284.8 eV was selected as the calibration positions for other peaks, including Mo 3*d*, O 1*s*, Ca 2*p*, P 2*p*, Ti 2*p*, and Zr 3*d*. XPS peak 4.1 software was used to fit and analyze the data. The pH value of Hank’s solution was monitored every 3 days using a pH meter (STARTER 3100, OHAUS, USA). The pH value of Hank’s solution without any samples was set as the control group.

To investigate the subsurface corrosion morphology beneath the corrosion layer, corrosion products were removed according to ASTM G1-03(2017) e1 ^[8]^. Samples were rinsed with deionized water, dried, and treated with 200 g/L CrO_3_ aqueous solution at 80 °C for 1 minute. The cleaned samples were then observed via optical microscopy and SEM for subsurface morphology. The corrosion rate was calculated using weight loss measurements before immersion and after corrosion layer removal, based on the equation:

$\text{P}_{\text{w}}\text{=8.74×}\text{10}^{\text{4}}\frac{\text{W}}{\text{A·t·}\text{ρ}}$ (Eq. 2)

Where *P_w_* is the corrosion rate (mm/year), W is the weight loss (g), A is the exposed area (cm²), t is the immersion time (hours), and *ρ* is the material density of Mo (10.28 g/cm^3^).

**1.4 *In-vitro* cell response**

*1.4.1 Cell culture*

Bone marrow mesenchymal stem cells (BMSCs, OriCell C57BL/6) and human umbilical vein endothelial cells (HUVECs, OriCell) were cultured under specific conditions. BMSCs were maintained in minimum essential medium-α (α-MEM; Hyclone, USA), while HUVECs were incubated in Dulbecco’s modified eagle medium (DMEM/F12; Hyclone, USA), both supplemented with 10% fetal bovine serum and 1% penicillin/streptomycin. The cells were incubated in a humidified environment at 37 ℃ with 5% CO_2_, and the culture media was replaced every 2 days.

*1.4.2 Direct cell culture*

HUVECs and BMSCs were seeded onto sample surfaces, with Ti6Al4V and AZ31 serving as control groups, and cultured for 1, 3, and 5 days. Note that Zn was included for comparison due to its relevance, but direct cell culture on Zn surfaces is not feasible because of its too high cytotoxicity ^[2, 9]^. Prior to cell seeding, the samples were sterilized under UV light for 1 hour and placed into 24-well plates. HUVECs or BMSCs were digested, resuspended, and seeded at a concentration of 2 × 10⁴ cells per mL onto the sample surfaces. At each time points (1, 3, and 5 days), the medium was removed, and the cells were washed three times with PBS. The cells were then fixed with 2.5% glutaraldehyde at room temperature for 4 hours, followed by three washes with PBS. The cells were then stained by rhodamine B to observe their proliferation and spreading. Briefly, 100 μL of diluted rhodamine B solution was added to the surface of the samples, and staining was carried out in the dark for 10 minutes. Following staining, the samples were observed under a fluorescence microscope (DMi8, Leica, Germany) with an exposure time of 1,000 ms to visualize cell morphology, and images were captured. The cell adhesion density and spread area were counted using ImageJ software (version 1.38e, National Institutes of Health, Bethesda, MD, USA). For SEM observations, the cells were fixed and dehydrated using an ethanol gradient (50%, 75%, 90%, and 100%) and subsequently, cells were imaged. At least three parallel samples were prepared and analyzed.

*1.4.3 Indirect cell culture*

Indirect cell culture was used to evaluate the cytotoxicity of Mo and Mo-Ti-Zr, and AZ31 was selected as the control group. Briefly, sample extracts were prepared in accordance with ISO 10993-5 (2009). The samples were sterilized under UV light for 1 hour and then immersed in cell culture medium containing 10% fetal bovine serum and 1% penicillin/streptomycin at a ratio of 1.25 cm^2^/mL. The samples were incubated at 37°C with 5% CO_2_ for 24 hours to obtain the extracts (100%). The concentrations of Mo and Zr element in the extract were measured using ICP-OES (Agilent ICP-OES 730, Agilent Technologies Inc., Wilmington, DE, USA). The sample extracts were diluted to 50% and 25% for cell culture. HUVECs and BMSCs were seeded in 96-well plates at a density of 5 × 10³ cells per well for 24 hours. The medium was then replaced with the extracts at 100%, 50%, and 25% concentrations. As controls, medium without extract was used as the negative control, and medium containing 10% dimethyl sulfoxide (DMSO; purity ≥ 99%, Sigma-Aldrich) was used as the positive control. After 1, 3, and 5 days of incubation, the medium was replaced with 200 μL of a medium containing 10% cell counting kit-8 (CCK-8) reagent (Sigma-Aldrich). Follwing incubation for an additional 3 hours, the CCK-8 solution was transferred to a new 96-well plate, and the optical density at 450 nm was measured using a microplate reader (Infinite F50, Tecan, Grodig, Austria). Cell viability was calculated using the following equation:

$\text{Cell viability (100\%) }\text{=}\text{ }\text{[(}\text{A}_{\text{s}}\text{-}\text{A}_{\text{p}}\text{)/(}\text{A}_{\text{n}}\text{-}\text{A}_{\text{p}}\text{)]}\text{×1}\text{00\%}$ (Eq. 3)

Where A_s_ ​is the optical density of the test samples, A_n_ is the optical density of the negative control, and A_p_ is the optical density of the positive control. At least three parallel samples were used.

Unless otherwise specified, subsequent in-vitro assays were performed using the 50% (v/v) extracts, because a preliminary screen with 100%, 50%, and 25% showed that Mo- and Mo–Ti–Zr extracts were non-cytotoxic at ≤50% whereas AZ31 was cytotoxic/suppressive at 100% and 50% but not at 25%; using 50% thus preserved cell viability for the Mo-based groups while providing a more discriminative ionic challenge than 25%.

For cell morphology observations, the cells were stained with rhodamine B (Sigma-Aldrich, St. Louis, MO, USA) and imaged using an inverted fluorescence microscope (Optiphot-2, Nikon, Tokyo, Japan) with a 600 ms exposure time, as mentioned in Section 1.4.2.

BMSCs and HUVECs were cultured with MoCl_5_ (Chendu Huaxia Huaxue Reagent Co., Ltd., Chengdu, China) at 0.1 (9.595 µg/mL), 0.2 (19.19 µg/mL), 0.5 (47.975 µg/mL), 1 (95.95 µg/mL), 2 (191.9 µg/mL), and 5 (479.75 µg/mL) mmol/L for 1, 3, and 5 days. Cell viability and rhodamine B staining were performed as mentioned above. Similarly, BMSCs and HUVECs were treated with ZrCl_4_ (Aladdin, Shanghai, China) at 0.05 (11.652 µg/mL), 0.1 (23.304 µg/mL), 0.25 (58.26 µg/mL), 0.5 (116.52 µg/mL), and 1 (233.04 µg/mL) mmol/L for 3 days. Cell viability was evaluated using the same procedure as described above, while live/dead staining was carried out to observe cell morphology. Furthermore, total RNA was extracted after 3 days of incubation, and RT-qPCR analysis was conducted to detect the expression of angiogenic and osteogenic markers, including CD31, VEGF, BMP-2, and Runx2.

**1.5 *In vitro* angiogenic activity assay**

*1.5.1 Cytoimmunofluorescence of HUVECs*

HUVECs were cultured in 50% concentration extracts of Mo and Mo-Ti-Zr for 3 days. Following this, cells were rinsed three times with PBS and fixed in 4% paraformaldehyde at room temperature for 15 minutes, and then permeabilized using 0.1% Triton X-100 for 20 minutes, washed twice with PBS, and blocked with goat serum for 30 minutes. Cells were incubated overnight at 4 °C with anti- vascular endothelial growth factor (VEGF, Abcam, Cat# ab46154, 1:200) and anti- cluster of differentiation 31 (CD31, Abcam, Cat# ab76533, 1:200) antibodies. After 24 hours, a secondary antibody (Invitrogen, Cat# A11008) was applied at room temperature for 1 hour, and the nuclei were stained with DAPI (Solarbio, China) for 5 min. Images were acquired using a confocal laser scanning microscope (Leica, STELLARIS 5, Heidelberg, Germany), and fluorescence intensity was analyzed quantitatively with ImageJ software.

*1.5.2 Real-time quantitative polymerase chain reaction (RT-qPCR) and western blotting (WB) assay*

RT-qPCR was performed to assess the expression of genes related to angiogenesis, including VEGF and CD31. The primer sequences used are provided in Table S6. HUVECs were cultured for 3 days, after which total RNA was extracted using the RNeasy Micro kit (Qiagen, Valencia, CA, USA) and subsequently treated with the Turbo DNA-Free kit (Life Technologies, Grand Island, NY, USA) to eliminate any residual DNA that could cause false-positive results. RNA quality and purity were verified with a spectrophotometer (Nanodrop, Thermo Fisher Scientific). Gene expression was quantified using the 2^−ΔΔCt^ method. Statistical analysis was conducted with data from at least four independent samples.

WB was performed to examine proteins involved in the Wnt–β/catenin signaling pathway, with GAPDH used as the loading control. Briefly, HUVECs were treated with a 50% sample extract for 3 days. Cells were then collected, suspended in PBS, incubated on ice for 30 minutes, and centrifuged at 12,000 rpm for 10 minutes. The supernatant, containing the total protein, was subjected to sodium dodecyl sulfate-polyacrylamide gel electrophoresis (SDS-PAGE) at 80 V for an initial 30 minutes, followed by 120 V for 1 hour using precast gels. The proteins were then transferred onto a methanol-activated PVDF membrane at 250 mA for 2.5 hours. Blocking was performed with Tris-buffered saline containing 0.05% Tween and 5% non-fat milk (Bio-Rad, Hercules, CA, USA), followed by overnight incubation at 4°C with primary antibodies against CD31 (Proteintech, Cat# 11265-1-AP), VEGF (Proteintech, Cat# 19003-1-AP), Wnt5a (Proteintech, Cat# 55236-1-AP), β-catenin (CST, Cat# 8480S), and GAPDH (CST, Cat# 2118), each diluted to 1:1,000 in TBST. The following day, the membrane was washed and incubated with a secondary antibody diluted to 1:5,000 in TBST for 1 hour at room temperature. Protein bands were detected using an Enhanced Chemiluminescence Kit (ECL; Simuwubio, Shanghai, China), and ImageJ software was used for quantification of relative protein expression levels.

**1.6 *In vitro* osteogenic activity assay**

*1.6.1 Cytoimmunofluorescence of BMSCs*

BMSCs were cultured and treated with extract media using a similar procedure to that described in Section 1.5.1 for HUVECs. After 3 days of incubation, cells were fixed with 4% paraformaldehyde for 15 minutes, permeabilized with 0.1% Triton X-100 for 20 minutes, and blocked with goat serum for 30 minutes. Cells were then incubated overnight at 4 °C with primary antibodies targeting osteogenesis-related proteins: anti-Osterix (OSX) (Abcam, Cat# ab22552, 1:200), anti-BMP-2 (Proteintech, Cat# 18933-1-AP, 1:200), and anti-Runx2 (Abcam, Cat# ab23981, 1:200). The next day, fluorescent secondary antibodies were applied for 1 hour at room temperature, followed by DAPI staining (Solarbio, China). Images were acquired using a confocal laser scanning microscope (Leica, STELLARIS 5, Germany), and fluorescence intensity was analyzed with ImageJ software.

*1.6.2 Alkaline phosphatase (ALP) and alizarin red (AR) assay*

For ALP staining, BMSCs (2 × 10^4^ in 1 mL medium) were seeded into each well of a 24-well plate. After 1 day of culture, the medium was replaced with a 50% sample extract containing β-glycerophosphate (1 mol/L), ascorbic acid (50 mmol/L), and dexamethasone (1 mmol/L), with control wells containing medium without extract. The medium was refreshed every 3 days. After 7 and 14 days, cells were fixed with 4% PFA for 20 minutes at room temperature, washed with PBS, and stained with BCIP/NBT alkaline phosphatase (Beyotime Biotechnology, Shanghai, China). Staining was observed under an optical microscope (MM6, Leitz Company, Germany) with a 1/50 s exposure and 3-mm focal length. For ALP activity analysis, after 7 and 14 days of culture, cells were rinsed with PBS, lysed with 1% Triton X-100 at 4 °C for 12 hours, and ALP activity in the lysate was measured using a kit (Nanjing Jiancheng Bioengineering Institute) following the manufacturer’s instructions. Total protein content was determined with a BCA protein assay kit, and ALP activity was normalized to protein concentration. Four samples were analyzed in parallel.

For AR staining and calcium nodule quantification, BMSCs were cultured as above. After 7 and 14 days, cells were fixed with 4% PFA, rinsed with deionized water, and stained with 200 μL AR solution (Solarbio Science & Technology, Beijing, China) for 20 minutes at room temperature. Cells were then washed and imaged under an optical microscope. For calcium quantification, cells were stained with 10% hexadecyl pyridinium chloride and absorbance at 562 nm was measured. Four replicates were used for statistical analysis.

*1.6.3 RT-qPCR and WB assay*

The procedures for RT-qPCR and WB were as described in Section 1.5.2. Osteogenesis-related genes, including *OSX*, *BMP-2*, and *Runx-2* were determined. For WB analysis, the following primary antibodies were used: anti-Runx2 (Abcam, Cat# ab23981), OSX (Abcam, Cat# ab22552), anti-OCN (Proteintech, Cat# 16157‑1‑AP), anti-BMP-2 (Proteintech, Cat# 18933-1-AP), anti-PKA (CST, Cat# 4782S), anti-phospho-PKA (CST, Cat# 5661S), anti-Akt (CST, Cat# 4691), anti-phospho-Akt (CST, Cat# 4060), anti-ERK (CST, Cat# 4695), anti-phospho-ERK (CST, Cat# 4370), GAPDH (CST, Cat# 2118) was used as the internal loading control.

*1.6.4 Transcriptome sequencing of BMSCs*

BMSCs (2 × 10⁵ cells) were seeded into a 6-well plate and incubated for 24 hours. The medium was then replaced with 50% extracts of Mo and Mo-Ti-Zr, while a group without extracts served as the control. After 3 days of culture, RNA was extracted using an RNA extraction kit (Axygen, AP-MN-MS-RNA-250, Corning, NY, USA) according to the manufacturer’s protocol. Eukaryotic mRNA was enriched with Oligo (dT)-conjugated magnetic beads and fragmented into shorter segments with an interrupting reagent. Single-stranded and double-stranded cDNA were synthesized using random six-base primers through a two-step reaction system. The double-stranded cDNA was then purified, end-repaired, and amplified by PCR to obtain specific fragment sizes. The resulting library was evaluated with an Agilent 2100 Bioanalyzer (Agilent Technologies Co., Ltd., Santa Clara, CA, USA) and sequenced on an Illumina platform. Pathway analysis and network construction were conducted using Ingenuity Pathway Analysis software (IPA^®^, v01-04, QIAGEN, Redwood City, CA, USA).

The identification and functional enrichment analysis of differentially expressed genes (DEGs) were as referenced in a previous study ^[10]^. For the gene ontology (GO) and kyoto encyclopedia of genes and genomes (KEGG) enrichment assay, the Z value was used as following equation:

$\text{Z}_{\text{score}}\text{=}\frac{\text{(Up-Down)}}{\sqrt{counts}}$ (Eq. 4)

**1.7 *In-vitro* evaluation of hemocompatibility**

*1.7.1 Coagulation time*

To investigate the coagulation time of Mo-Ti-Zr and Mo, the clotting time, including the activated partial thromboplastin time (APTT), prothrombin time (PT), and thrombin time (TT), were determined according to our previous study ^[11]^. In brief, a mixed solution of 200 µL of poor platelet plasma (PPP) and 100 µL of actin-activated cephaloplastin reagent was pipetted onto the sample surface, followed by the addition of 100 µL CaCl_2_ (0.03 M) and incubation for 30 minutes at 37 °C. The APTT was then assessed. For the measurements of TT, 200 µL of PPP was pipetted onto the surface of samples for 30 minutes at 37 °C. PPP was then collected and added into the TT reagent (200 µL, Shanghai Sun Biotech Co. Ltd., Shanghai, China) in a test tube to assess the TT. Similarly, 200 µL of PPP was mixed with 400 µL of PT reagent (Shanghai Sun Biotech Co. Ltd., Shanghai, China) to assess the PT. At least four samples were used in parallel for each group.

*1.7.2 Hemolysis rate*

The hemolysis rate of Mo-Ti-Zr was compared with Mo. Fresh blood was obtained from a volunteer (approved by the ethics committee of Southwest Jiaotong University) and mixed with 0.9% NaCl solution at a blood-to-NaCl volume ration of 4:5. The specimens were then soaked with 10 mL of 0.9% NaCl solution in centrifuge tube at 37 °C for 30 min. Then 200 µL of the mixed solution was added to the tube and incubated for a further 60 minutes. Subsequently, the samples were retrieved, and the solutions were transferred into new tubes and centrifuged at 1,000 rpm for 5 minutes. The optical density of the supernatant was detected by a microplate reader (Infinite F50, Tecan, Grodig, Austria). The hemolysis rate of the samples was calculated according to the formula:

$\text{Hemolysis rate (100\%) }\text{=}\text{ }\text{[(}\text{A}_{\text{s}}\text{-}\text{A}_{\text{p}}\text{)/(}\text{A}_{\text{n}}\text{-}\text{A}_{\text{p}}\text{)]}\text{×1}\text{00\%}$ (Eq. 5)

Where A_s_ is the absorbency of samples, A_p_ and A_n_ represent the optical densities of positive and negative control groups, respectively.

Note the 0.9% NaCl solution without samples and deionized water were selected as the negative and positive control groups, respectively. Four samples were analyzed in parallel used for the detection of hemolysis rate.

**1.8 *In vivo* animal study**

*1.8.1 Preparation of Mo and Mo-Ti-Zr-based intramedullary nails (IMNs)*

Mo and Mo-Ti-Zr IMNs with a diameter of 1.5 ± 0.05 mm and a length of 25 ± 0.01 mm were employed for the animal experiments (Figure S4, Supplementary Materials). All IMNs, including the Mo-Ti-Zr, Mo, stainless steel (SS; modified from standard clinical Kirschner wires) and pure Zn groups for comparison, were fabricated by the following same procedure: the bar materials were machined first to the shape and size as above described, and then subjected to the same surface treatment procedure, namely a sequence of mechanical polishing (from 600 up to 2000# grit) , ultrasonic cleaning with deionized water, ethanol, and acetone, before all samples were sterilized in 70% ethanol for 30 minutes followed by ultraviolet (UV-C) irradiation for 30 minutes on each side. These consistent procedures ensured comparable surface conditions across all groups. It should be noted that neither pure Zn nor pure Mg are ideal for use as IMNs in load-bearing bone due to their relatively low mechanical strength and rapid degradation—Mg degrades too fast, while Zn is prone to brittle fracture during prolonged implantation ^[12, 13]^. Nonetheless, pure Zn was selected here as a relatively better, widely studied and representative biodegradable metal to provide a reference baseline for *in vivo* performance comparison ^[14, 15]^. However, for cytotoxicity Zn ions has much lower tolerance than Mg ions and we selected Mg alloy (AZ31 is the most investigated) for *in vitro* bio-assays.

*1.8.2 Femur fracture model establishment and in vivo surgery*

All animal procedures were conducted with approval from the Institutional Animal Care and Use Committee of Peking University Shenzhen Hospital (Ethical Permit Number: 2022-164), adhering to the guidelines of the Chinese Council on Animal Care. A total of 48 female Sprague-Dawley rats (8 weeks old, weighing approximately 200–220 g), sourced from Guangdong Medical Laboratory Animal Center (Guangzhou, China), were used for this study.

To induce femur bone fractures, osteotomies were performed. Specifically, rats were anesthetized with isoflurane (Ante Animal Husbandry Technology Co., Ltd., Jinan, China) using a gas anesthesia system (MSS-3, Keighley, UK). The hairs on the left leg of rats were shaved and a 2-cm incision was made to access the mid-femur. A medical wheel saw was employed to create a transverse fracture at the mid-shaft, after which stabilization was achieved by inserting an unlocked Kirschner wire as an intramedullary pin (no bicortical interlocking), the standard fixation method in rat femoral fracture models. Rats were divided into four groups: SS (n=12), pure Zn (n=12), Mo (n=12), and Mo-Ti-Zr (n=12). Following implantation, the incision was sutured, and each rat received an intraperitoneal injection of buprenorphine (0.3 mg/kg, Temgesic, Reckitt & Cloman, Hull, UK) for pain relief and ceftriaxone (100 mg/kg, Aladdin) as an antibiotic. At 24-hours post-surgery, an X-ray radiograph of the operative site was taken using the Amadea V-Drmini (OR Technology, Germany) with settings of 55 kV, 3.2 mA, and a 10 ms exposure time.

At 6 and 12 weeks after surgery, the rats were anesthetized with isoflurane and X-rays of the implanted leg were taken to assess fracture healing using a scoring system (Table S8, Supplementary Materials). After the designated time points, the animals were euthanized, and the implanted femurs along with other organs (brain, heart, liver, spleen, lung, and kidney) were harvested and fixed in 10% formalin. Each femur was scanned using the micro-computed tomography system (μCT 80; SCANCO Medical AG, Switzerland) with a voltage of 70 kV, current of 114 μA, integration time of 250 ms, and a resolution of 15.6 μm. Bone parameters such as bone mineral density (BMD), bone volume to total volume (BV/TV), trabecular number (Tb. N), trabecular thickness (Tb. Th), and trabecular separation (Tb. Sp) were measured in the area around the fracture (approximately 0.5 mm in length). Following fixation, the femurs were decalcified by immersion in 10% ethylenediaminetetraacetic acid solution for 2 months. Decalcified femurs were then embedded in paraffin and sectioned into 5-μm slices for hematoxylin & eosin (H&E), Masson’s trichrome, Goldner’s, and immunohistochemical staining. Immunohistochemical analyses targeted AKT, BMP-2, COL I, and CD31. Imaging was performed using a high-resolution microscope (Olympus Co., Ltd.). The organs, including the brain, heart, liver, spleen, lungs, and kidneys, were collected and weighed. For H&E staining, the organs were embedded in paraffin, sectioned into 5-μm slices, stained, and examined. To evaluate the Mo concentration, each organ was digested using 2 mL of HNO_3_ (analytical grade, Chengdu Kelong Chemical Co., Ltd.) and 0.5 mL of H_2_O_2_ (analytical grade, ≥30% purity, Chengdu Kelong Chemical Co., Ltd.) for 15 minutes, followed by heating at 90°C for 100 minutes. In addition, peri-fracture tissues within approximately 1 mm around the implant were collected and processed using the same digestion protocol to determine both Mo and Zr concentrations. The concentrations of Mo and Zr in the digested samples were determined using inductively coupled plasma mass spectrometry (ICP-MS; PerkinElmer NexION 300X).

*1.8.3 In vivo degradation behavior of IMNs*

Prior to decalcification, IMNs were carefully extracted, rinsed three times with deionized water, and dried in a vacuum oven (Tianjin Taisite Instrument Co., Ltd., Tianjin, China). Surface morphology was observed using the SEM and elemental composition was determined via EDS as mentioned above. To remove corrosion products, the method described in Section 1.3.2 was followed, after which the IMNs were re-examined by SEM. The weights of the IMNs were recorded both before implantation and after removal of corrosion products to calculate the corrosion rate as described in Eq. 2 in Section 1.3.2.

*1.8.4 In vivo mechanical evolution of IMNs*

The mechanical properties of Mo and Mo-Ti-Zr IMNs before and after implantation were evaluated using a three-point bending test. Cylindrical samples (diameter: 1.5 mm; length: 25 mm) were tested using a mechanical testing machine (AG-IS, Shimadzu, Japan) equipped with a custom jig at a crosshead speed of 5 mm/min. IMNs were retrieved from rats at 6 and 12 weeks post-implantation, and then subjected to bending tests to assess changes in implant mechanical integrity. The maximum bending force (N) was recorded at fracture. The maximum bending stress 𝜎 was calculated using the following equation:

$\text{σ=}\frac{\text{3}\text{FL}}{\text{2}\text{bd}\text{2}}$ (Eq. 6)

where *F* is the maximum load (N), *L* is the span length (17.61 mm), *b* is the width of the cylindrical sample (1.5 mm), and *d* is the height or diameter (1.5 mm).

Due to the limited length of the intramedullary nails (25 mm), a span length of 17.61 mm was adopted, which slightly deviates from the standard L/d ratio. However, this configuration is suitable and consistent for miniature implant testing, and allows sufficient support without end effects interfering with the central loading region.

**1.9 Statistical analysis**

All experiments were repeated at least three times independently. The quantitative data are presented as mean ± standard deviation (SD). SPSS Statistics 24.0 (IBM Corp, Armonk, NY, USA) was utilized to analyze the data. Student’s *t*-test was used to compare the differences between two groups, and one-way analysis of variance (ANOVA) was used to compare the difference between three or more groups. A two-tailed p-value < 0.05 was considered statistically significant. In the figures, asterisks indicate the p-value: *p < 0.05, **p < 0.01, and ***p < 0.001.

1. **Supporting Images**


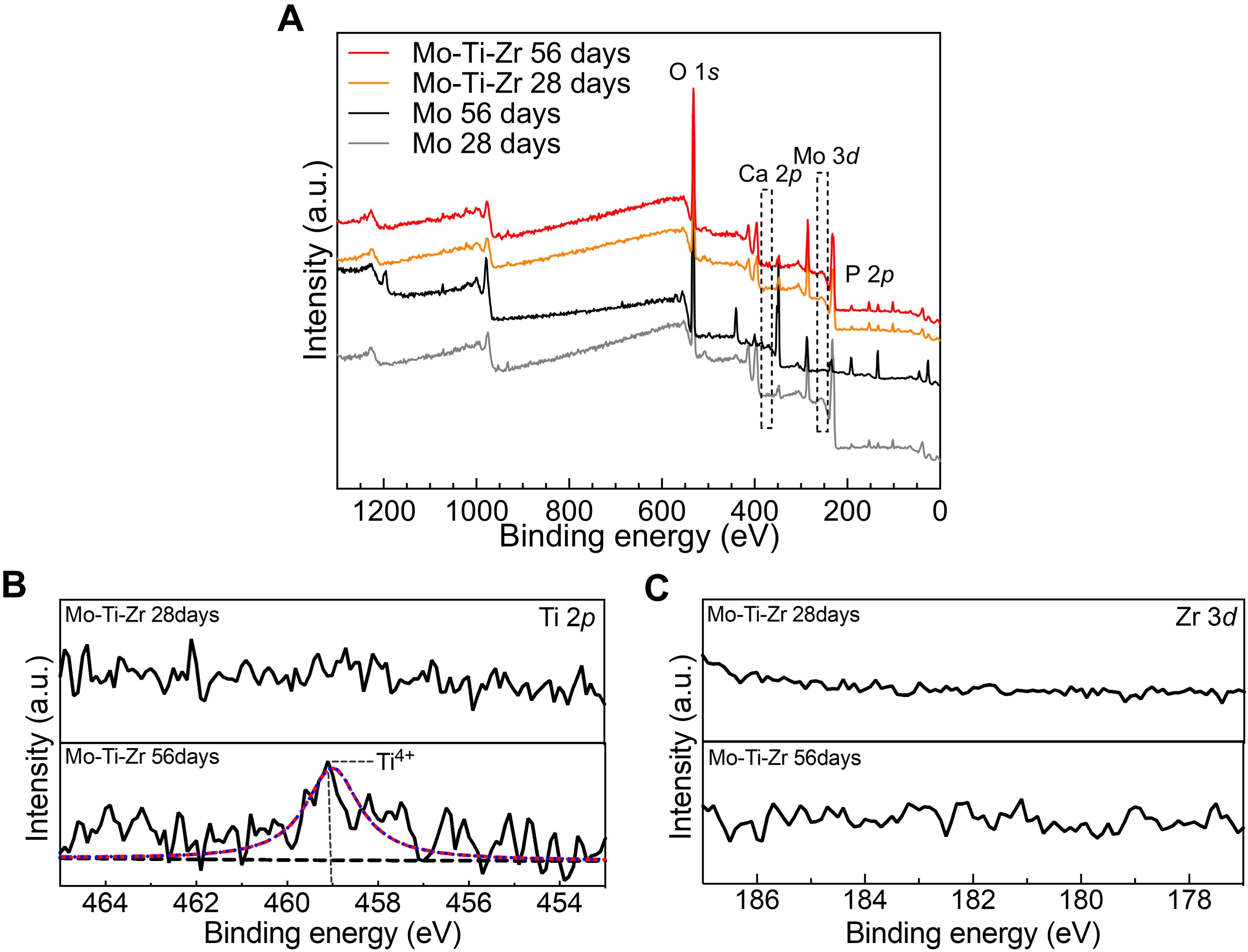


**Figure S1.** (A) X-ray photoelectron spectroscopy (XPS) survey of Mo-Ti-Zr compared with Mo after 28 and 56 days of immersion in Hank’s solution at 37 °C. (B) and (C) High-resolution XPS of Ti 2*p* and Zr 3*d*, respectively.





**Figure S2.** (A) Representative live/dead staining images of HUVECs cultured with different concentrations of ZrCl_4_ (5-1000 μM) for 3 days (live cells: green; dead cells: red; scale bar: 200 μm). (B) Quantitative analysis of cell viability. (C, D) RT-qPCR analysis of angiogenic markers of CD31 and VEGF, respectively. Data are presented as mean ± SD (n = 3); *p < 0.05, **p < 0.01, ***p < 0.001.





**Figure S3.** (A) Representative live/dead staining images of BMSCs cultured with different concentrations of ZrCl_4_ (5-1000 μM) for 3 days (live cells: green; dead cells: red; scale bar: 200 μm). (B) Quantitative analysis of cell viability. (C, D) RT-qPCR analysis of angiogenic markers of Runx2 and BMP-2, respectively. Data are presented as mean ± SD (n = 3); *p < 0.05, **p < 0.01, ***p < 0.001.


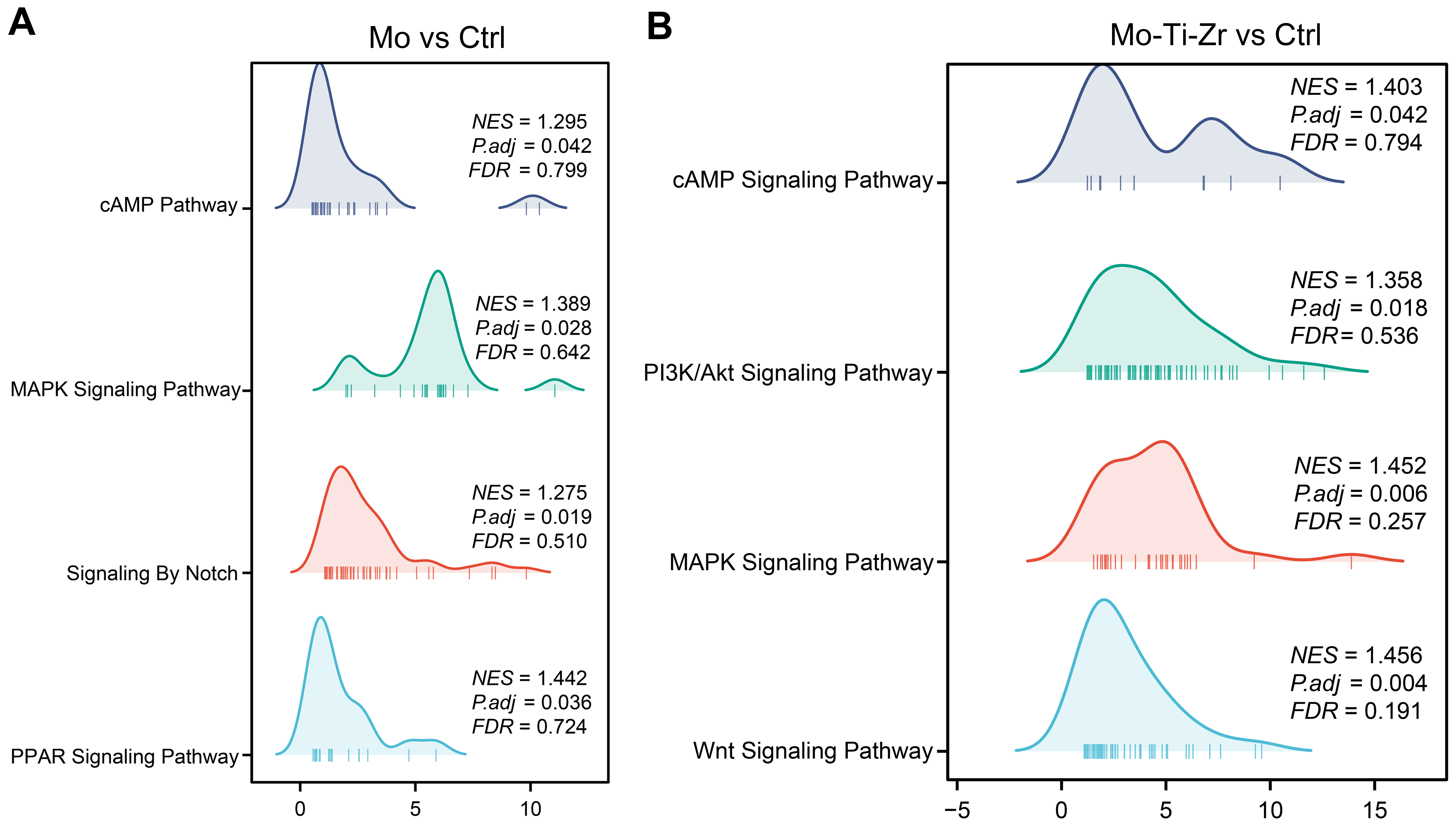


**Figure S4.** Transcription sequencing of BMSCs cultured with the extraction of Mo and Mo-Ti-Zr. (A1-A2) GSEA enrichment plots of the positive regulatory signaling pathways of Mo vs. Ctrl and Mo-Ti-Zr vs. Ctrl.


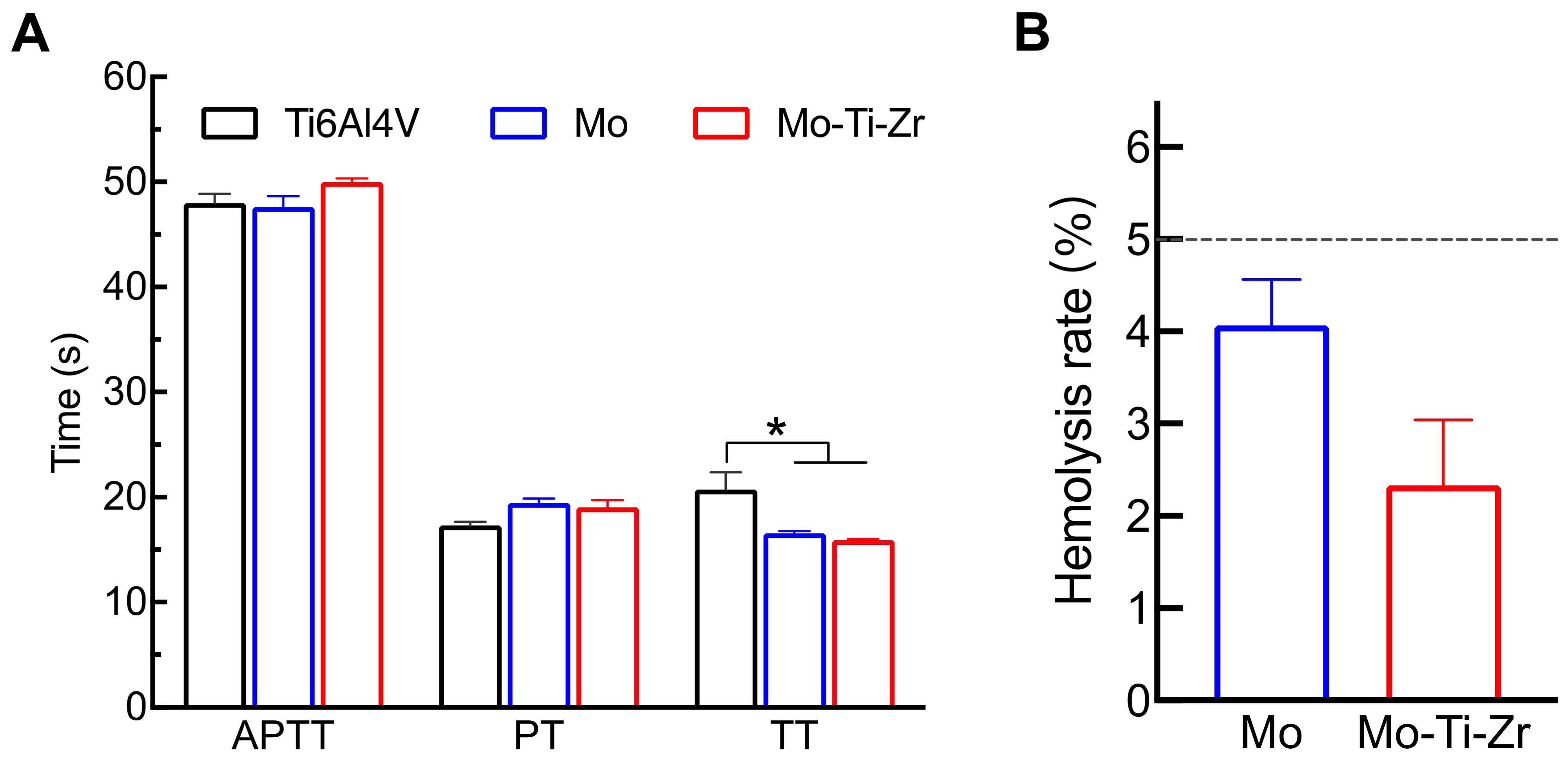


**Figure S5.** Hemocompatibility of Mo-Ti-Zr compared to Mo and SS. (A) Activated partial thromboplastin time (APTT), prothrombin time (PT), and thrombin time (TT). (B) Hemolysis rate.


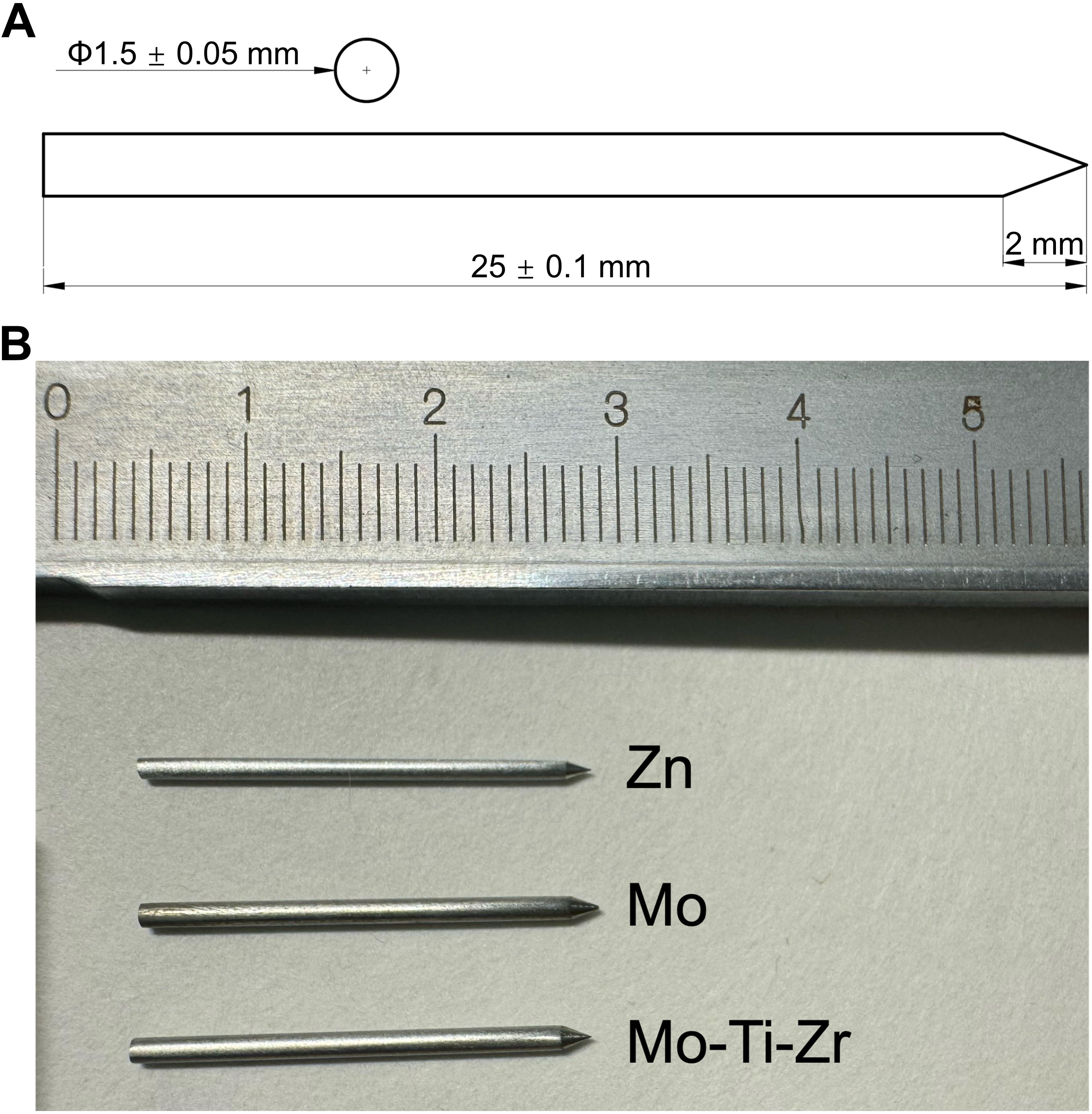


**Figure S6.** (A) Schematic illustration of the intramedullary nails (IMNs) for rats’ femur implantation. (B) Optical images of Mo-Ti-Zr compared with Mo and pure Zn-based IMNs.





**Figure S7.** Ion concentrations in peri-fracture tissues. (A) Zr concentration (µg/L). (B) Mo concentration (mg/kg) in the surrounding tissues after implantation of SS, Zn, Mo, and Mo–Ti–Zr intramedullary nails. Data are presented as mean ± SD.

1. **Supporting Tables**

**Table S1.** The parameters obtained from transient electrochemical measurements, including corrosion potential (*E*_corr_), current density (*i*_corr_), and corrosion rate (*P*_i_), EIS spectra fitting results of Mo-Ti-Zr compared with Mo.

| Samples | Parameters obtained from PDP curves | | | The fitted values of the parameters of EIS spectra | | | |
| --- | --- | --- | --- | --- | --- | --- | --- |
|  | *E*_corr_  (V_SCE_) | *i*_corr_  (μA⋅cm^-2^) | *P*_i_  (μm/year) | *Q*_p_×10^-4^  (S^n^Ω^-1^cm^-2^) | *R*_p_  (Ωcm^2^) | *Q*_ct_×10^-4^  (S^n^Ω^-1^cm^-2^) | *R*_ct_  (Ωcm^2^) |
| Mo | -0.40 ± 0.03 | 3.17 ± 0.27 | 16.14 ± 1.37 | 1.12 ± 0.31 | 397.00 ± 11.13 | 3.02 ± 0.33 | 7.43 ± 0.17 |
| Mo-Ti-Zr | -0.35 ± 0.02 | 2.81 ± 0.46 | 14.30 ± 2.34 | 67.98 ± 0.28 | 68.60 ± 8.21 | 1.41 ± 0.41 | 9.89 ± 0.11 |

**Table S2.** The EIS fitting results of Mo-Ti-Zr compared with Mo after 7, 14, 28, and 56 days of immersion in Hank’s solution at 37 °C.

| Samples | The fitted values of the parameters of EIS spectra | | | | | |
| --- | --- | --- | --- | --- | --- | --- |
|  | *R*_s_  (Ωcm^2^) | *Q*_p1_×10^-3^  (S^n^Ω^-1^cm^-2^) | *R*_p_  (Ωcm^2^) | *Q*_ct_×10^-3^  (S^n^Ω^-1^cm^-2^) | *R*_ct_  (Ωcm^2^) | *Z_w_*  (s^1/2^·Ω^-1^·cm^-2^) |
| Mo |  |  |  |  |  |  |
| 7day | 10.89 ± 0.18 | 3.03 ± 0.03 | 25.43 ± 7.14 | 4.63 ± 0.07 | 8.40 ± 0.24 | 46.38 ± 0.54 |
| 14day | 14.24 ± 0.11 | 2.30 ± 0.02 | 726.60 ± 6.81 | 0.85 ± 0.11 | 1.05 ± 0.11 | 65.49 ± 0.61 |
| 28day | 7.87 ± 0.08 | 45.82 ± 0.04 | 1637.00 ± 8.21 | 0.34 ± 0.03 | 13.35 ± 0.19 | 47.18 ± 0.38 |
| 56day | 7.54 ± 0.09 | 0.06 ± 0.01 | 2.27 ± 0.02 | 1.38 ± 0.04 | 0.401 ± 0.03 | 45.10 ± 0.91 |
| Mo-Ti-Zr |  |  |  |  |  |  |
| 7day | 14.24 ± 0.13 | 2.32 ± 0.07 | 786.60 ± 11.26 | 0.87 ± 0.05 | 9.44 ± 0.27 | 65.49 ± 0.23 |
| 14day | 13.98 ± 0.15 | 3.39 ± 0.09 | 649.70 ± 13.82 | 1.16 ± 0.12 | 10.72 ± 0.24 | 54.79 ± 0.73 |
| 28day | 7.30 ± 0.08 | 9.62 ± 0.05 | 918.20 ± 8.25 | 0.35 ± 0.02 | 13.69 ± 0.13 | 25.13 ± 0.68 |
| 56day | 7.06 ± 0.12 | 1.63 ± 0.02 | 4.16 ± 0.21 | 8.08 ± 0.32 | 282.50 ± 1.68 | 807.20 ± 3.59 |

**Table S3.** Elements composition of Mo-Ti-Zr compared to Mo after 28 and 56 days of immersion in Hank’s solution at 37 °C obtained from energy dispersive spectroscopy.

| Samples | Weight composition (wt%) | | | | |
| --- | --- | --- | --- | --- | --- |
|  | Mo | O | C | Ca | P |
| Mo 28-days | 69.2 | 21.0 | 7.6 | 1.2 | 1.0 |
| Mo 56-days | 62.8 | 26.5 | 5.9 | 2.7 | 2.1 |
| Mo-Ti-Zr 28-days | 68.8 | 22.3 | 6.9 | 1.4 | 0.65 |
| Mo-Ti-Zr 56-days | 66.3 | 24.2 | 6.2 | 1.7 | 1.6 |

**Table S4.** Atomic composition of Mo-Ti-Zr compared to Mo after 28 and 56 days of immersion in Hank’s solution at 37 °C obtained from XPS spectra.

| Samples | Atomic Composition (%) | | | | | | |
| --- | --- | --- | --- | --- | --- | --- | --- |
|  | Mo | O | C | Ca | P | Ti | Zr |
| Mo 28days | 9.91 | 43.27 | 41.93 | 2.27 | 2.62 | - | - |
| Mo 56days | 3.31 | 51.25 | 23.07 | 12.31 | 10.06 | - | - |
| Mo-Ti-Zr 28days | 6.04 | 39.38 | 47.4 | 2.36 | 3.07 | 1.33 | 0.42 |
| Mo-Ti-Zr 56days | 3.82 | 46.26 | 41.39 | 2.50 | 4.23 | 1.37 | 0.43 |

**Table S5.** Mechanical properties of Mo-Ti-Zr compared with Mo ^[16]^.

| Materials | Tensile strength (MPa) | Yield strength (MPa) | Breaking elongation (%) | Hardness |
| --- | --- | --- | --- | --- |
| Mo | 457 ± 23 | 396 ± 15 | 11.3 ± 0.4 | 234 ± 13 |
| Mo-Ti-Zr | 747 ± 31 | 643 ± 11 | 13 ± 1.2 | 235 ± 9 |

**Table S6.** Primer sequences of genes in relation to angiogenesis of HUVECs cells.

| Real-time quantitative PCR primer sequence | |
| --- | --- |
| Gene | Sequence (5’- 3’ on minus strand) |
| *GAPDH* | Fwd: GTCTCCTCTGACTTCAACAGCG |
|  | Rev: ACCACCCTGTTGCTGTAGCCAA |
| *VEGF* | Fwd: TTGCCTTGCTGCTCTACCTCCA |
|  | Rev: GATGGCAGTAGCTGCGCTGATA |
| *CD31* | Fwd: AACAGTGTTGACATGAAGAGCC |
|  | Rev: TGTAAAACAGCACGTCATCCTT |

**Table S7.** Primer sequences of genes in relation to osteogenesis of BMSC cells.

| Real-time quantitative PCR primer sequence | |
| --- | --- |
| Gene | Sequence (5’- 3’ on minus strand) |
| *GAPDH* | Fwd: AGGTCGGTGTGAACGGATTTG |
|  | Rev: TGTAGACCATGTAGTTGAGGTCA |
| *Runx2* | Fwd: AACGATCTGAGATTTGTGGGC |
|  | Rev: CCTGCGTGGGATTTCTTGGTT |
| *ALP* | Fwd: CCAACTCTTTTGTGCCAGAGA |
|  | Rev: GGCTACATTGGTGTTGAGCTTTT |
| *OCN* | Fwd: GAGGGCAATAAGGTAGTGAA |
|  | Rev: CCATAGATGCGTTTGTAGGC |
| *OPN* | Fwd: AGCAAGAAACTCTTCCAAGCAA |
|  | Rev: GTGAGATTCGTCAGATTCATCCG |
| *OSX* | Fwd: GTCCTCTCTGCTTGAGGAA |
|  | Rev: CTTGAGAAGGGAGCTGGGT |

**Table S8.** Grading system for femoral shaft fracture healing.

| Grade | Description | Score |
| --- | --- | --- |
| 1 | No calcification | 1 |
| 2 | Patchy calcification | 2 |
| 3 | Calcification has the appearance of a callus | 3 |
| 4 | Callus bridging the fracture gap | 4 |
| 5 | Continuity of bone trabeculae | 5 |
| 6 | Remodeling to normal bone | 6 |

**References**

[1] C. Pöhl, J. Schatte, H. Leitner, Metallographic characterization of the molybdenum based alloy MHC by a color etching technique, Materials Characterization 77 (2013) 63-69.

[2] Y. Su, K. Wang, J. Gao, Y. Yang, Y.-X. Qin, Y. Zheng, D. Zhu, Enhanced cytocompatibility and antibacterial property of zinc phosphate coating on biodegradable zinc materials, Acta Biomaterialia 98 (2019) 174-185.

[3] L. Zhu, X. Tong, Z. Ye, Z. Lin, T. Zhou, S. Huang, Y. Li, J. Lin, C. Wen, J. Ma, Zinc phosphate, zinc oxide, and their dual-phase coatings on pure Zn foam with good corrosion resistance, cytocompatibility, and antibacterial ability for potential biodegradable bone-implant applications, Chemical Engineering Journal 450 (2022) 137946.

[4] Y. Chen, W. Zhang, M.F. Maitz, M. Chen, H. Zhang, J. Mao, Y. Zhao, N. Huang, G. Wan, Comparative corrosion behavior of Zn with Fe and Mg in the course of immersion degradation in phosphate buffered saline, Corrosion Science 111 (2016) 541-555.

[5] F. Gao, E. Su, J. Hou, J. Wang, Y. Zhou, H. Qin, Z. Xie, J. Mao, H. Li, W. Tao, Progressively advantageous long-term corrosion profile of Mo over Mg, Zn, and Fe from the perspective of biodegradable metals, Corrosion Science 240 (2024) 112439.

[6] M. Sikora-Jasinska, L.M. Morath, M.P. Kwesiga, M.E. Plank, A.L. Nelson, A.A. Oliver, M.L. Bocks, R.J. Guillory II, J. Goldman, In-vivo evaluation of molybdenum as bioabsorbable stent candidate, Bioactive Materials 14 (2022) 262-271.

[7] STANDARD ASTM, G31-72, Standard practice for laboratory immersion corrosion

testing of metals, ASTM International, West Conshohocken, PA, 2004.

[8] ASTM G1-03, 2017e1Standard practice for preparing, cleaning, and evaluating

corrosion test dpecimens, ASTM International, West Conshohocken, PA2017.

[9] J. Qian, H. Qin, P. Zeng, J. Hou, X. Mo, G. Shen, H. Zeng, W. Zhang, Y. Chen, G. Wan, Metal-organic Zn-zoledronic acid and 1-hydroxyethylidene-1, 1-diphosphonic acid nanostick-mediated zinc phosphate hybrid coating on biodegradable Zn for osteoporotic fracture healing implants, Acta Biomaterialia 166 (2023) 685-704.

[10] H. Qin, W. Sheng, G. Zhang, Q. Yang, S. Yao, Y. Yue, P. Zhang, Y. Zhu, Q. Wang, Y. Chen, Comprehensive analysis of cuproptosis-related prognostic gene signature and tumor immune microenvironment in HCC, Frontiers in Genetics 14 (2023) 1094793.

[11] J. Qian, Z. Zhang, F. Gao, J. He, J. Hou, E. Su, Z. Zeng, W. Wang, Y. Chen, W. Zhang, Titanium dioxide covalently immobilized citric Acid (TiO_2_‐CA) nanohybrid coating of neurovascular flow diverter to improve antithrombogenic and pro‐endothelialization properties, Advanced Materials Interfaces 10(5) (2023) 2202197.

[12] K. Xie, L. Wang, Y. Guo, S. Zhao, Y. Yang, D. Dong, W. Ding, K. Dai, W. Gong, G. Yuan, Effectiveness and safety of biodegradable Mg-Nd-Zn-Zr alloy screws for the treatment of medial malleolar fractures, Journal of Orthopaedic Translation 27 (2021) 96-100.

[13] Z. Zhang, B. Jia, H. Yang, Y. Han, Q. Wu, K. Dai, Y. Zheng, Zn0.8Li0.1Sr—a biodegradable metal with high mechanical strength comparable to pure Ti for the treatment of osteoporotic bone fractures: in vitro and in vivo studies, Biomaterials 275 (2021) 120905.

[14] J. Qian, H. Qin, P. Zeng, J. Hou, X. Mo, G. Shen, H. Zeng, W. Zhang, Y. Chen, G. Wan, Metal-organic Zn-zoledronic acid and 1-hydroxyethylidene-1, 1-diphosphonic acid nanostick-mediated zinc phosphate hybrid coating on biodegradable Zn for osteoporotic fracture healing implants, Acta Biomaterialia 166 (2023) 685-704.

[15] J. Qian, H. Qin, E. Su, J. Hou, H. Zeng, T. Wang, D. Wang, G. Wan, Y. Chen, Angiogenesis-osteogenesis coupling and anti-osteoclastogenesis zoledronate intermixed calcium silicate metal-organic/inorganic hybrid coating on biodegradable zinc-based intramedullary nails for osteoporotic fracture healing, Bioactive Materials 44 (2025) 46-67.

[16] B. Cockeram, The mechanical properties and fracture mechanisms of wrought low carbon arc cast (LCAC), molybdenum-0.5 pct titanium-0.1 pct zirconium (TZM), and oxide dispersion strengthened (ODS) molybdenum flat products, Materials Science Engineering A 418(2006) 120-136.
